# E-cigarette exposure triggers distal lung cell injury, persistent lung stress response, and antiviral immune suppression

**DOI:** 10.1172/jci.insight.198757

**Published:** 2026-08-11

**Authors:** Tanner C. Rivera, Kelly S. Schweitzer, Christina Cornell, Jordan Nall, Nicholas Egersdorf, Courtney Moeder, Riley A. Cooney, Eszter K. Vladar, Steve D. Groshong, Gregory P. Downey, James P. Bridges, Richard Bowen, Hong Wei Chu, Irina Petrache

**Affiliations:** 1 Department of Medicine, Division of Pulmonary and Critical Care Medicine, National Jewish Health, Denver, Colorado.; 2 Department of Medicine, Division of Pulmonary Sciences and Critical Care Medicine, University of Colorado, Denver, Colorado, USA.; 3 Department of Biomedical Sciences, Colorado State University, Fort Collins, Colorado, USA.

**Keywords:** Pulmonology, Vascular biology, Autophagy, Cell stress, Respiration

## Abstract

The mechanisms by which e-cigarette vaping (EV) affects lung health remain unclear. Clusters of EV-associated lung injury indicate that EV damages distal lung parenchyma and increases vulnerability to second-hit injury, including respiratory viral infections. Using human lung endothelial and epithelial cells and precision-cut lung slices, we investigated the mechanisms underlying distal lung cell injury and repair triggered by brief (24-hour) EV exposure. RNA-seq of lung tissue from golden Syrian hamsters evaluated the persistence of lung stress responses (10 days after 5 days of EV exposure) and the effect of EV on host defense against influenza A virus and SARS-CoV-2. EV disrupted the barrier function of human distal lung cells through JNK stress response signaling, triggered autophagy with impaired flux, suppressed mTOR signaling and cell proliferation, and culminated in apoptosis. Transcriptional responses in EV-exposed hamster lungs revealed persistent activation of JNK signaling, autophagy, barrier dysfunction, tissue remodeling, and impaired Th1 immunity. EV increased SARS-CoV-2 viral burden, downregulated antiviral genes (Ifit1, Isg15, Nfkbia), and amplified oxidative stress and IL-12 signaling. Short-term EV exposure triggered stress-induced distal lung cell injury with persistent changes in antiviral immunity and molecular pathways associated with tissue remodeling. That may increase susceptibility to viral infections and contribute to lung disease.

## Abstract 2

Short-term e-cigarette use damages lung cells, impairs repair, and weakens antiviral defenses, raising concerns about long-term health risks.

## Introduction

Electronic cigarette (e-cigarette) vaping (EV) has emerged as a popular alternative to tobacco smoking (1, 2). EV poses important risks to lung health (3, 4), most dramatically reflected in a cluster of cases of acute respiratory failure from EV product use–associated lung injury (EVALI) (5), which was attributed to concurrent EV use with vitamin E or tetrahydrocannabinol or infection with respiratory viruses (5, 6). This suggests that EV predisposes to a second-hit injury of the distal lung, which may include weakening of the epithelial and endothelial lung barriers, thereby inducing acute lung inflammation and edema. Additionally, subtle repetitive lung barrier dysfunction has been implicated in chronic obstructive pulmonary disease (COPD) (7, 8) and pulmonary fibrosis (9, 10). We have shown that both nicotine-containing and nicotine-free EV weakens the lung endothelial cell barrier (11) through unknown mechanisms. EV exposure of primary human small airway epithelial cell (hSAEC) cultures and human precision-cut lung slices (PCLSs) triggers an exaggerated inflammatory response to viruses, weakening antiviral signaling (12, 13). EV has been shown to increase angiotensin-converting enzyme 2 (ACE2) levels, particularly in male mice (14), which could increase the susceptibility to coronavirus infection. These reports indicate that EV has deleterious effects on the host response to subsequent hits such as respiratory viral infections.

Stress-induced signaling pathways, including c-Jun N-terminal kinase (JNK), are critical regulators of cellular responses to environmental insults like reactive oxygen species or aldehydes that are present in EV (11). JNK signaling has been linked to increased endothelial and epithelial permeability, impaired repair, cell death, autophagy (15, 16), and inflammation. Although autophagy is a protective mechanism during stress, dysregulated autophagy may cause cell death and barrier dysfunction (17). Prior studies implicating JNK in autophagy in cigarette smoke–exposed cells suggest a role in EV-induced lung injury (17).

To better understand the acute and chronic effects of EV, most investigations relied on laboratory mice, revealing increased oxidative stress (11, 18), emphysema-like lung remodeling (19, 20), and extrapulmonary fibrosis (21). Further, most studies of EV in human specimens have focused on proximal airways (21). Additional animal modeling, complemented with studies in human distal lung specimens, is essential for bridging knowledge gaps. We study here golden Syrian hamsters (*Mesocricetus auratus*) because of their human-resembling small airway architecture (22) and inflammatory responses to respiratory viruses, including severe acute respiratory syndrome coronavirus 2 (SARS-CoV-2) (23). Hamsters respond to acute EV exposure by upregulating airway epithelial inflammatory gene and oxidative stress responses (24), rendering it optimal to study EV effects on lung barriers and responses to influenza A virus (IAV) and SARS-COV-2.

We show that EV triggers a JNK-mediated stress response that impairs distal lung barrier function and autophagy, with effects that persist even after cessation of EV exposure, manifesting as alterations in immune and tissue remodeling pathways that change host responses to acute respiratory viral infections.

## Results

### Barrier dysfunction of endothelial and epithelial monolayers following EV exposure.

**Figure 1:**
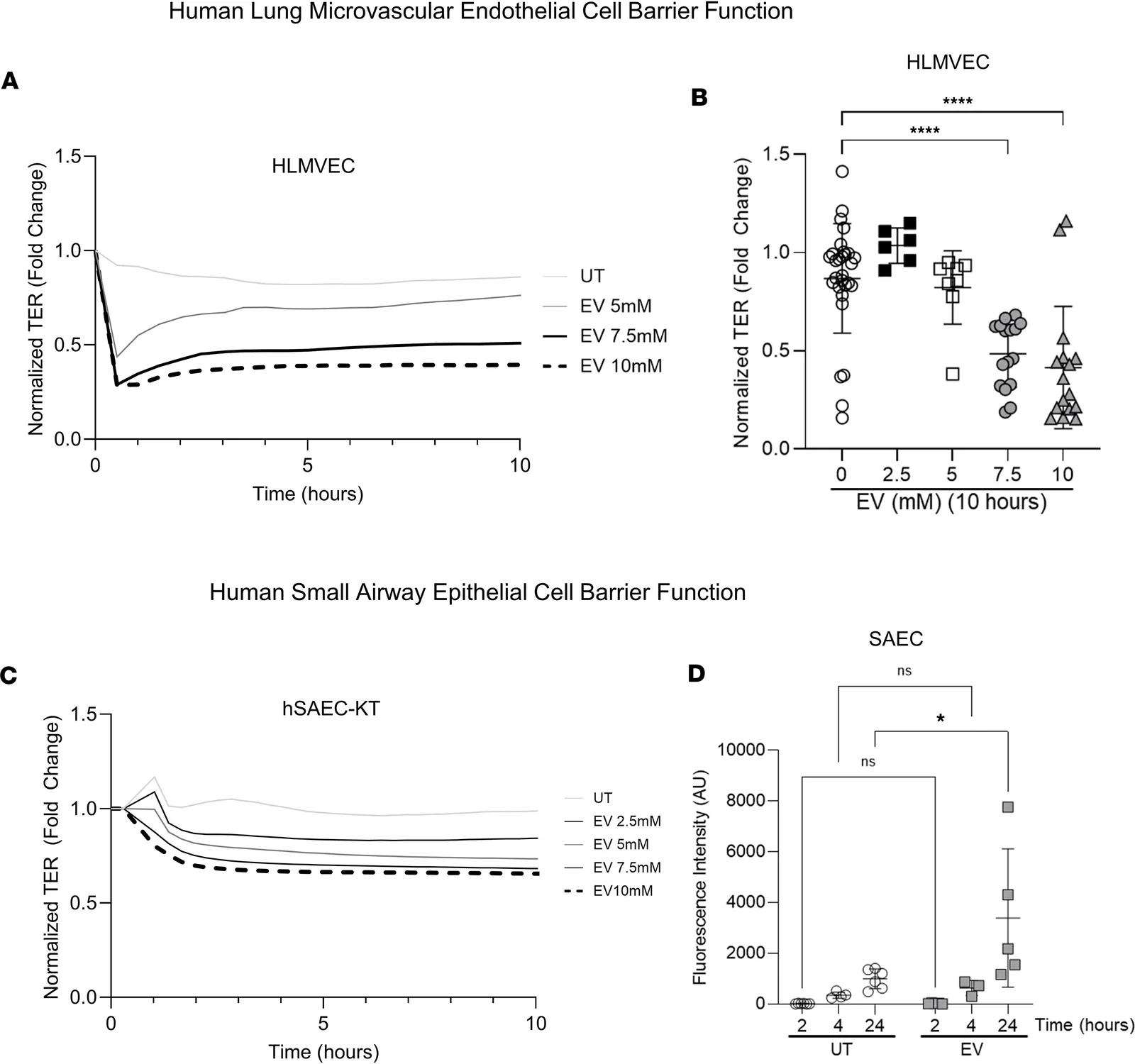
Barrier function of endothelial and epithelial monolayers following EV exposure. (**A** and **C**) Representative tracings of kinetics of trans-endothelial/epithelial electrical resistance (TER), normalized and expressed as fold change versus initial (prior to exposure), of primary human lung microvascular endothelial cells (HLMVECs; **A**) and transformed human small airway epithelial cells (hSAEC1-KT; **C**) exposed to EV at the indicated concentrations. (**B**) Scatterplot of normalized TER measured in HLMVECs exposed to EV for 10 hours. Mean ± SEM; symbols are individual biological replicates; 1-way ANOVA with Tukey’s test (*n* = 6–28, ****P < 0.0001). (**D**) Scatterplot of fluorescence intensity, measured in arbitrary units (AU) in the basolateral chamber of FITC-labeled dextran (4 kDa) apically applied to a primary hSAEC pseudostratified layer grown on a Transwell to air-liquid interface for 21 days and exposed to EV (7.5 mM) for the indicated times. Mean ± SEM; symbols are individual biological replicates; 1-way ANOVA (*n* = 6–29, *P < 0.05). UT, untreated.

Trans-endothelial/epithelial electrical resistance (TEER, or TER) measured by electric cell-substrate impedance sensing (ECIS) and transcellular dextran permeability measured by fluorimetry were recorded over time following EV addition. In primary human lung microvascular endothelial cells (HLMVECs), exposure to EV resulted in a dose-dependent reduction in barrier function (Figure 1A). When normalized to before exposure, TER decreased immediately following EV concentrations (5 mM, 7.5 mM, and 10 mM) that simulate casual vaping in humans, with the 10 mM concentration showing the most profound and sustained effect (Figure 1B). EV impaired hSAEC-KT (25) barrier function in a dose-dependent manner (Figure 1C), with concentrations of 7.5 mM and 10 mM showing the greatest effect. In primary hSAECs differentiated into a pseudostratified layer at the air-liquid interface for 3 weeks, EVs reduced barrier function, as assessed by FITC-labeled dextran transmigration (Figure 1D). The slower kinetics compared with those measured by ECIS in the submerged cells likely reflect differences in assay methodology and sensitivity.

### Autophagy in lung endothelial and epithelial cells following EV exposure.

**Figure 2:**
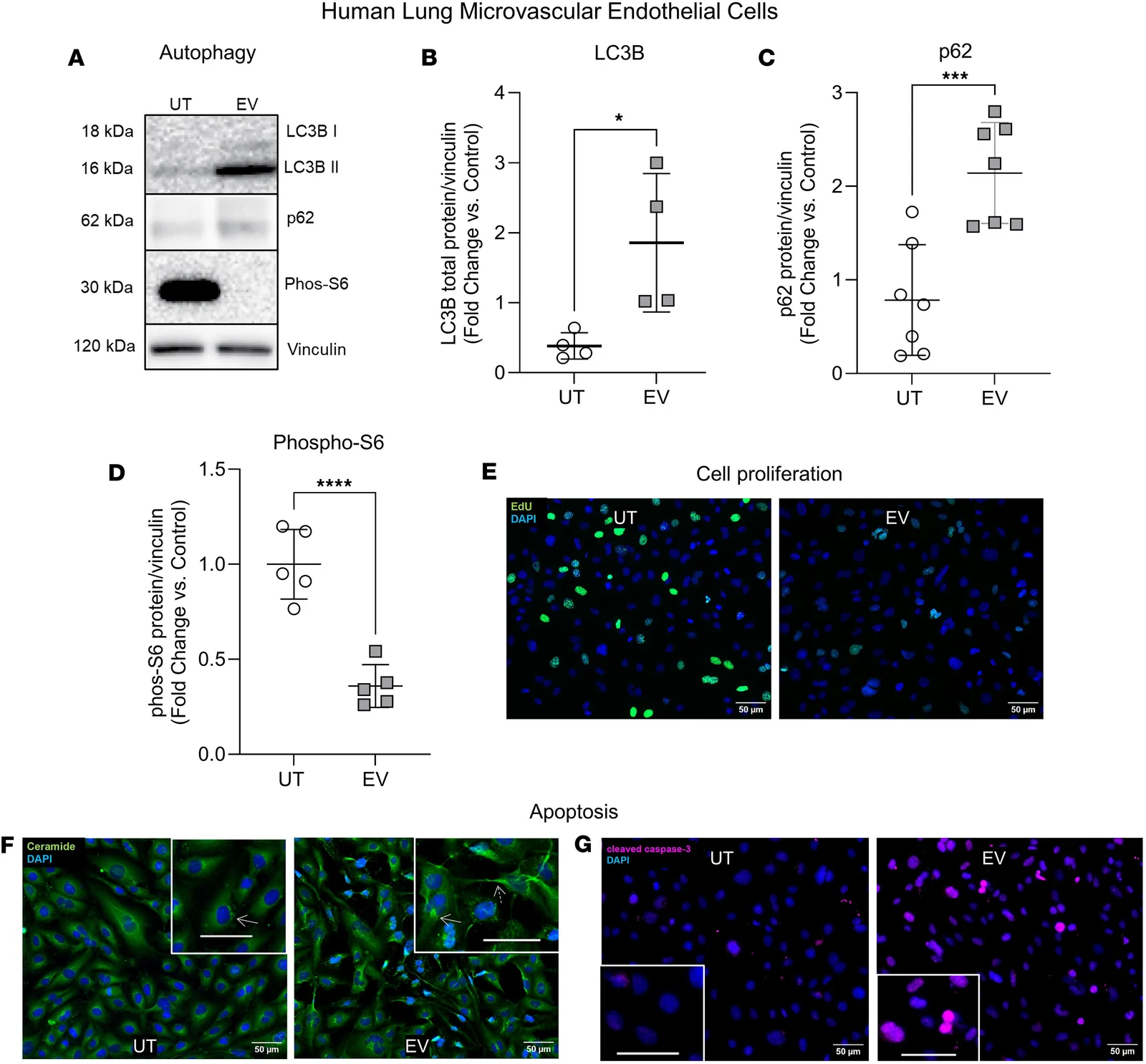
Effect of EV exposure on HLMVEC autophagy. (**A**) Representative Western blots of LC3B-II, p62/SQSTM1, and phosphorylated S6 in HLMVECs exposed to EV (7.5 mM, 24 hours). (**B**–**D**) Scatterplots of respective protein abundance measured by densitometry and expressed relative to vinculin loading control. Mean ± SEM; symbols are individual biological replicates; unpaired *t* test (**B**, *n* = 4, *P = 0.03; **C**, *n* = 7, ***P = 0.002; **D**, *n* = 5, ****P < 0.0001). (**E**–**G**) Representative immunofluorescence image of cells exposed to EV (7.5 mM, 24 hours) and stained for nuclei with DAPI (blue) and for incorporated fluorescently labeled EdU (green; **E**), ceramide (green; **F**), or cleaved caspase-3 (magenta; **G**). UT, untreated. Note that EV-exposed cells showed decreased proliferation; increased ceramide abundance predominantly localized perinuclearly (solid arrows in insets) and in plasma membrane (dashed arrow in inset); and increased cleaved caspase-3 abundance with almost exclusive nuclear localization (inset). Scale bars: 50 μm.

Next, we determined the effect of EVs on repair via stress-induced autophagy. In HLMVECs, EV exposure increased levels of microtubule-associated protein-1 light chain 3β (LC3B-II), with a punctate appearance on immunofluorescence imaging, indicating autophagosome formation (Figure 2, A and B, and Supplemental Figure 1A; supplemental material available online with this article; https://doi.org/10.1172/jci.insight.198757DS1), and p62/sequestosome 1 (SQSTM1) (Figure 2, A and C, and Supplemental Figure 1B), consistent with autophagy without full autophagolysosomal degradation. Phospho–ribosomal protein S6 (phospho-S6) was reduced by EV (Figure 2D), indicating suppressed mechanistic target of rapamycin (mTOR), consistent with induction of autophagy. EV reduced cell proliferation, as indicated by decreased EdU staining (Figure 2E). EV increased ceramide levels in plasma membranes and perinuclear regions and increased the cleaved caspase-3 translocation to the nucleus (Figure 2, F and G), consistent with apoptotic signaling.

**Figure 3:**
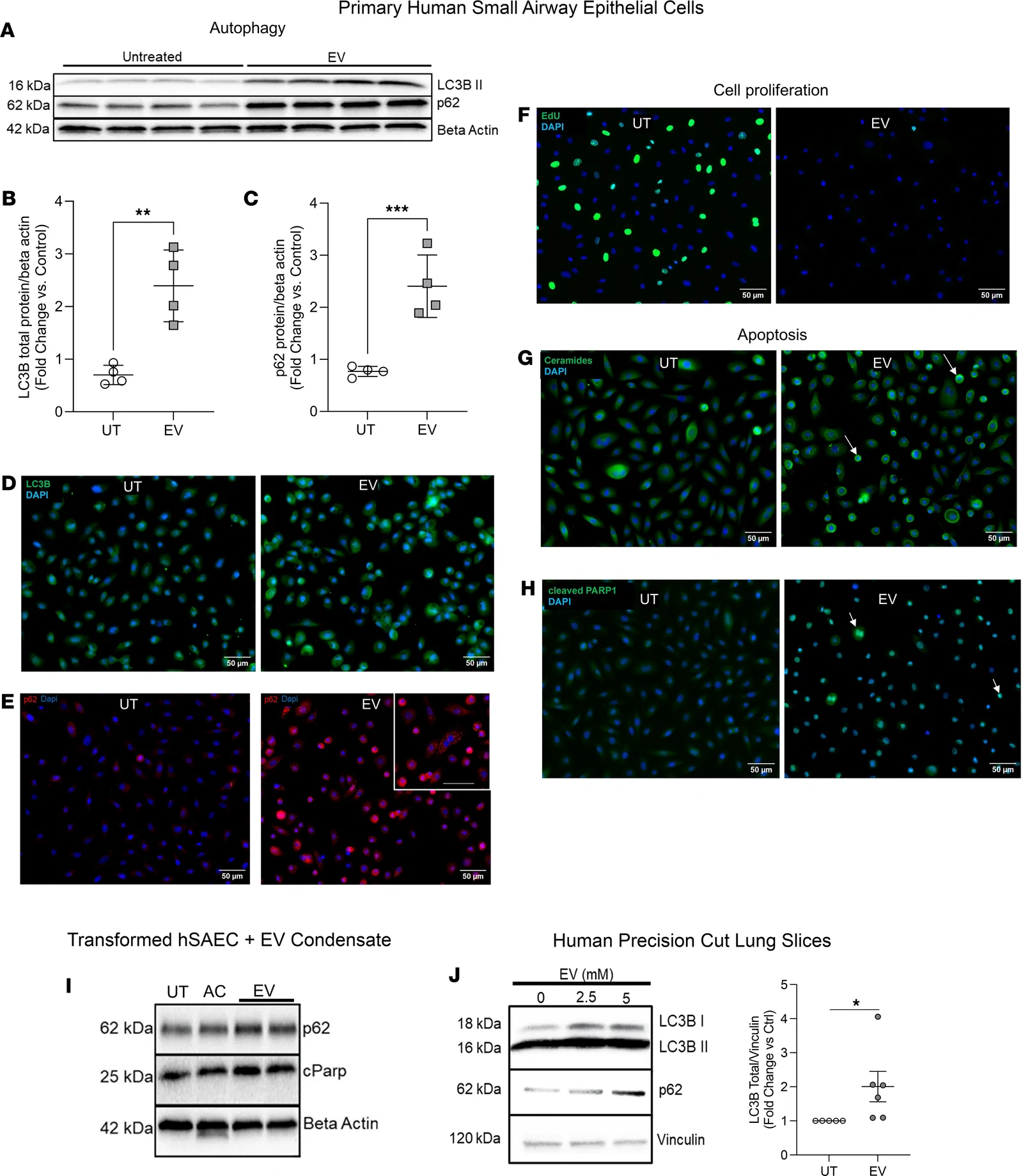
Effect of EV exposure on human lung small airway epithelial cell autophagy. (**A**) Representative Western blots of LC3B-II and p62 in hSAEC1-KT exposed to EV (7.5 mM, 24 hours). (**B** and **C**) Scatterplots of respective protein abundance measured by densitometry and expressed relative to β-actin loading control. Mean ± SEM; symbols are individual biological replicates; unpaired *t* test (**B**, *n* = 4, **P = 0.003; **C**, *n* = 4, ***P < 0.001). (**D**–**H**) Representative immunofluorescence microscopy images of hSAEC1-KT exposed to EV (7.5 mM, 24 hours) and stained for nuclei with DAPI (blue) and for LC3B (green; **D**), p62 (red; **E**), incorporated fluorescently labeled EdU (green; **F**), ceramides (**G**), and cleaved PARP (green; **H**). (**I**) Representative Western blots of p62 and cleaved PARP (cPARP) with actin as loading control in hSAEC1 cells exposed to EV condensate or ambient air condensate (5%; 24 hours, *n* = 3). UT, untreated. (**J**) Representative Western blots of LC3B-I/II and p62 with vinculin as loading control in human precision-cut lung slices exposed to EV at the indicated concentrations (*n* = 5) with relative changes measured by densitometry shown as fold change vs. UT (* *P* < 0.05). Scale bars: 50 μm.

In hSAEC1-KT, EV increased LC3B-II and p62/SQSTM1 levels (Figure 3, A–E) and markedly decreased cell proliferation, measured by EdU (Figure 3F). EV increased apoptosis, as indicated by increased ceramide staining at the plasma membrane (Figure 3G) and increased cleaved PARP1, cell rounding, and nuclear condensation (Figure 3H). Similar responses were observed in cells exposed to EV juice condensate (Figure 3I) and in human PCLSs exposed to EV (Figure 3, J and K).

### JNK inhibition improves barrier function and autophagy completion in EV-exposed HLMVECs.

**Figure 4:**
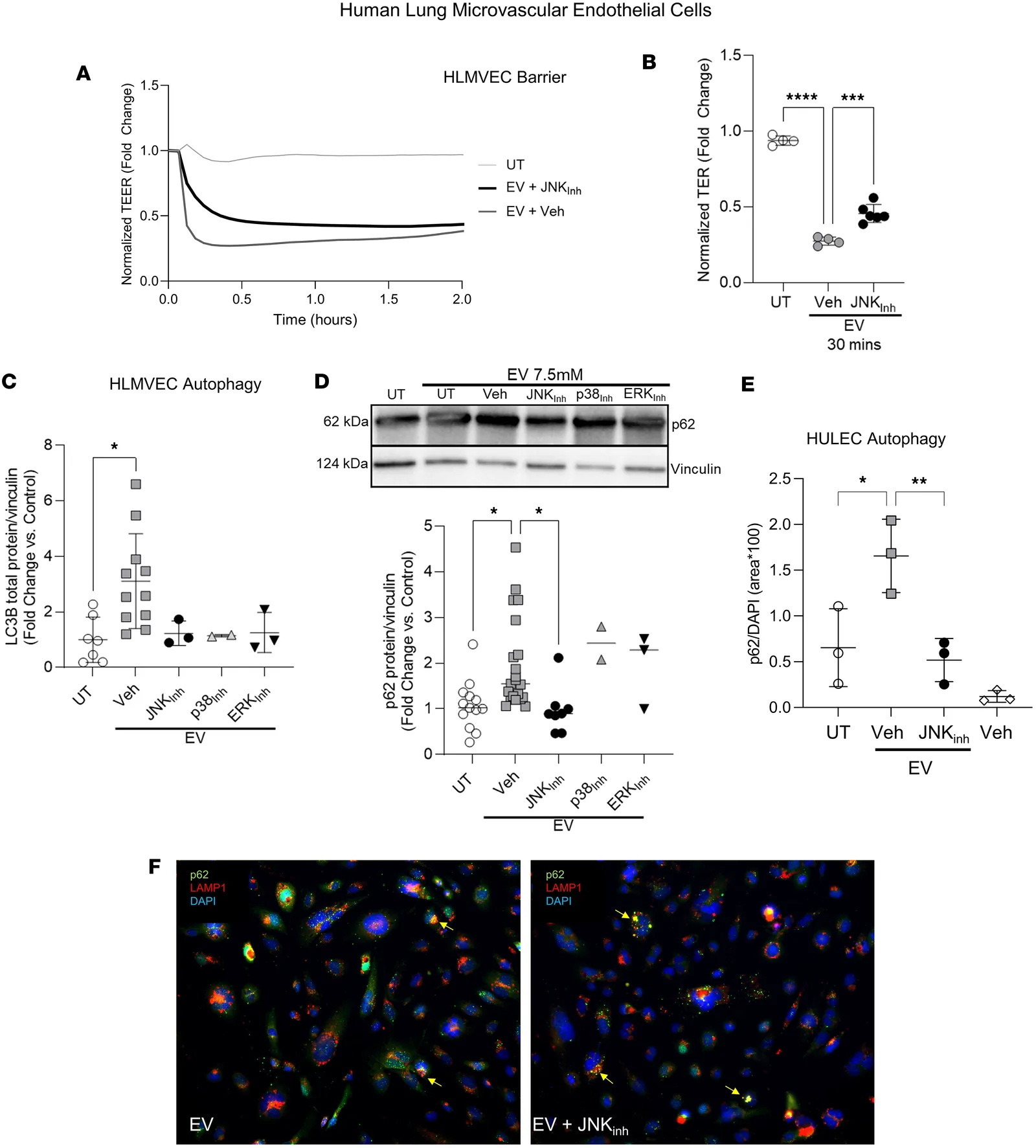
Role of JNK in EV-induced human lung microvascular endothelial autophagy. (**A**) Representative tracings of kinetics of normalized TER expressed as fold change versus initial measurement in HLMVECs exposed to EV (7.5 mM) in the presence of JNK inhibitor (JNK_inh_; SP600125, 50 μM) or its vehicle (Veh; DMSO, 1%). (**B**) Scatterplot of normalized TER measured in HLMVECs exposed to EV (7.5 mM, 30 minutes) in the presence of JNK_inh_ or Veh. Mean ± SEM; symbols are individual biological replicates; 1-way ANOVA, Tukey’s test (*n* = 4–6, ***P < 0.001, ****P < 0.0001). (**C** and **D**) Scatterplot of densitometry of LC3B-II (**C**) and p62 (**D**) relative to vinculin loading control in HLMVECs exposed to EV (7.5 mM, 24 hours) in the presence of JNK_inh_, p38 MAPK inhibitor (p38_inh_; SB202190, 30 μM), ERK inhibitor (ERK_inh_; PD98059, 50 μM), or Veh. Mean ± SEM; 1-way ANOVA, Tukey’s test (**C**, *n* = 2–11, *P = 0.03; **D**, *n* = 2–22, *P = 0.01). Representative Western blot of p62 and vinculin is shown in **D**. (**E**) Scatterplot of relative abundance of p62 relative to DAPI detected by immunofluorescence (IF) microscopy and measured by blinded automatic image analysis, in HULECs exposed to EV (7.5 mM, 24 hours) in the presence of Veh or JNK_inh_. Mean ± SEM; 2-way ANOVA, Tukey’s test (*n* = 3, *P < 0.05, **P < 0.01). (**F**) Representative IF images of HULECs exposed to EV in the presence of vehicle (left) or JNK_inh_ (right), immunostained for p62/SQSTM1 (green), lysosomes (LAMP1, red), and nuclei (DAPI, blue). Yellow arrows indicate superimposed p62 and LAMP1 staining (yellow) suggesting autophagolysosome fusion events. Original magnification ×40. UT, untreated.

To determine the mechanism by which EV triggers incomplete autophagy and barrier dysfunction, we next interrogated the MAPK pathways. In HLMVECs, under conditions of decreased barrier integrity induced by EV (7.5 mM), pretreatment with a pan-JNK inhibitor (SP600125, 50 μM) significantly attenuated this effect (Figure 4, A and B), indicating that JNK contributes to barrier disruption. Western blotting showed that JNK inhibition significantly reduced EV-induced LC3B and p62/SQSTM1 levels (Figure 4, C and D). Complementary immunofluorescence microscopy showed that transformed HLMVECs pretreated with a JNK inhibitor, compared with vehicle, had reduced accumulation of p62/SQSTM1 (Figure 4, E and F). Co-immunostaining for p62/SQSTM1 and the lysosomal marker LAMP1 (Figure 4F) revealed that in EV-exposed cells, most p62-stained puncta did not colocalize with lysosomes (indicating reduced autophagolysosomal fusion). While JNK inhibition reduced the abundance of p62-stained puncta, it had no effect on LAMP1-stained lysosome abundance, suggesting that JNK does not impact lysosomal biogenesis. The EV-induced increase in LC3B-II was significantly reduced by inhibitors of JNK, p38 MAPK, or ERK MAPK pathways (Figure 4C), whereas only JNK inhibition reduced EV-induced p62/SQSTM1 accumulation (Figure 4D). Therefore, JNK signaling is a critical mediator of both barrier dysfunction and autophagy dysregulation, whereas multiple MAPK pathways are involved in EV-triggered autophagosome formation.

### JNK inhibition attenuates barrier dysfunction and autophagy in EV-exposed hSAECs.

**Figure 5:**
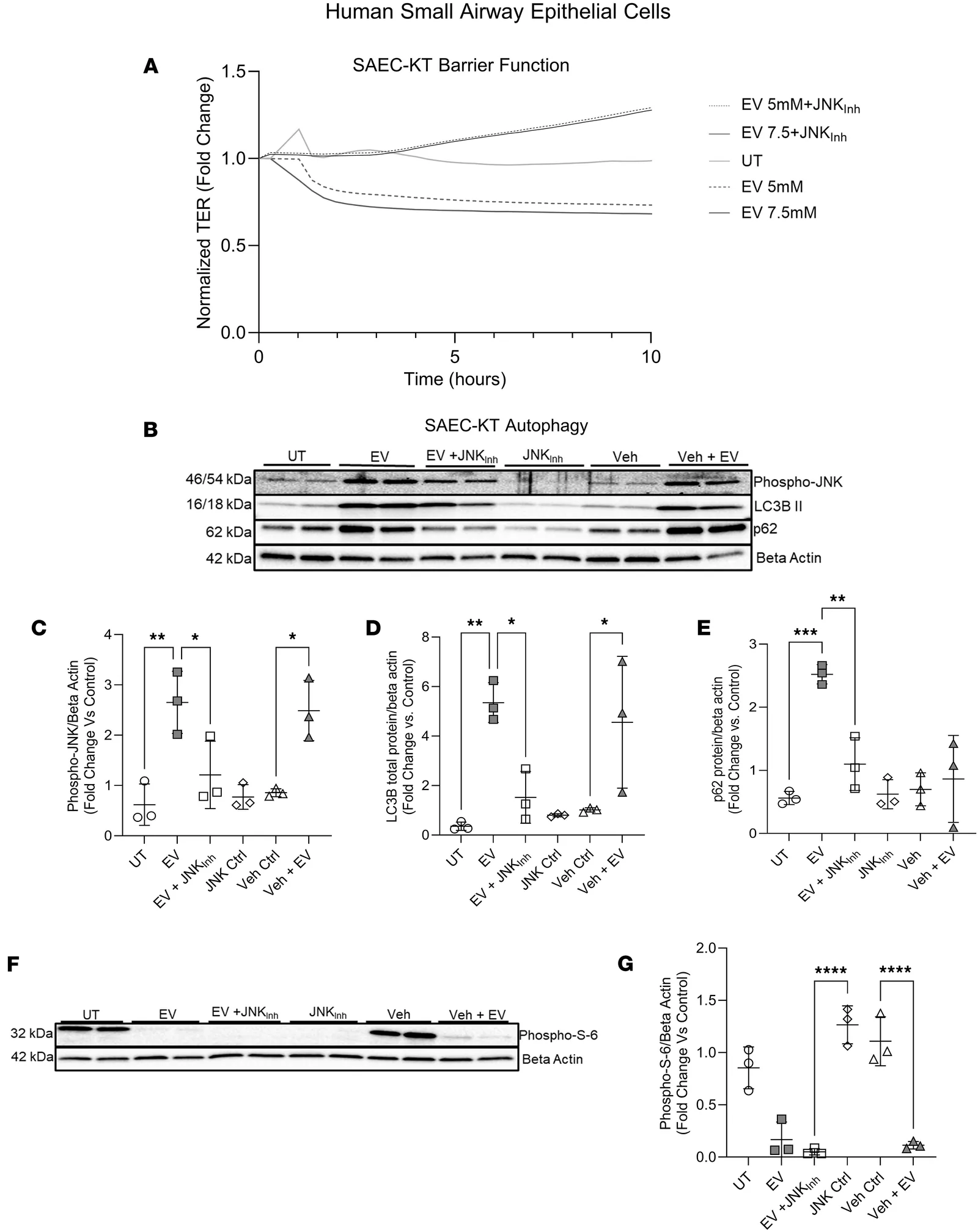
Role of JNK in EV-induced human lung small airway epithelial autophagy. (**A**) Representative tracings of kinetics of TER normalized and expressed as fold change versus initial measurement in hSAEC1-KT exposed to EV (5 or 7.5 mM) in the presence of JNK_inh_ (SP600125; 50 μM) or vehicle (DMSO). (**B**–**E**) Representative Western blots (**B**) and respective densitometries of phospho-JNK (**C**), LC3B-II (**D**), and p62 (**E**) relative to β-actin (as loading control) in hSAEC1-KT exposed to EV (5 or 7.5 mM, 24 hours) in the presence of JNK_inh_ (SP600125; 50 μM) or vehicle. (**F** and **G**) Representative Western blots (**F**) and respective densitometries of phospho-S6 (**G**) relative to β-actin. (**C**–**E** and **G**) Mean ± SEM; symbols are individual biological replicates (*n* = 3 in each, 1-way ANOVA, Tukey’s test, *P < 0.05, ** *P* < 0.01, ***P < 0.001, ****P < 0.0001). UT, untreated.

EV-induced barrier disruption in hSAEC1-KT was attenuated by a pan-JNK inhibitor (Figure 5A). Confirming that EV induced JNK activation, immunoblotting and immunofluorescence demonstrated increased phospho-JNK levels (Figure 5, B and C, and Supplemental Figure 1C). The EV-triggered accumulation of LC3B-II and p62/SQSTM1 was reduced by JNK inhibition (Figure 5, B, D, and E), indicating improved autophagy completion. EV reduced phospho-S6, indicative of mTOR inhibition regardless of JNK inhibition (Figure 5, F and G), implying that JNK acts downstream or independently of mTOR in regulating the completion of autophagy.

### Short-term EV exposure causes persistent cellular stress responses in hamster lungs.

**Figure 6:**
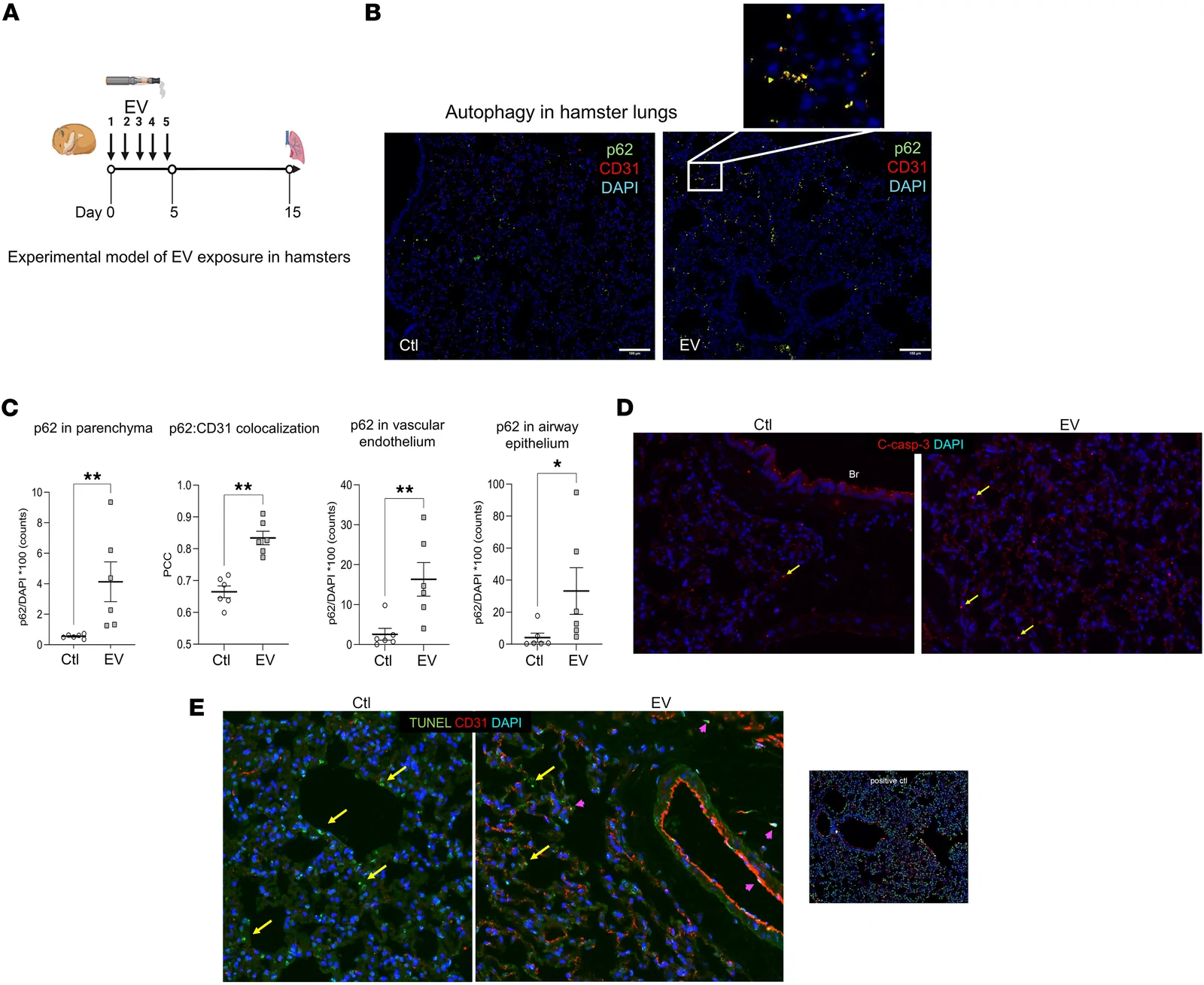
Persistent effects of brief EV exposure on distal lung autophagy in golden Syrian hamsters. (**A**) Schematic of experimental design depicting daily EV exposure via inhalation for 5 consecutive days, followed by removal from exposure for 10 days and harvest at day 15. (**B**) Representative immunofluorescence images of EV-exposed hamster lung parenchyma stained for nuclei (DAPI, blue), autophagy marker p62/SQSTM1 (green), and endothelial cell marker CD31 (red) (scale bar size: 100 µm; inset: 50x magnification). (**C**) Respective scatterplots of abundance of p62 in the parenchyma, including endothelial p62-CD31 cell colocalization (PCC) scores; in the vascular endothelium; and in the airway epithelium. *P < 0.05; **P < 0.01. (**D** and **E**) Representative immunofluorescence images of EV-exposed hamster lung parenchyma stained for nuclei (DAPI, blue) and cleaved caspase-3 (red; yellow arrows), ×10 magnification. (**D**) or DNA strand break marker TUNEL (green; yellow arrows) and endothelial cell marker CD31 (red), ×20 magnification (**E**). Note the colocalization of TUNEL/CD31 in EV-exposed lung parenchyma (magenta arrowheads). As a positive control, lung slides were treated with DNase.

To gain further insights into repair processes and potential long-term consequences of acute EV exposure, hamsters were exposed to EV aerosols at concentrations shown to simulate human exposure (26) via 5 consecutive daily nasal nebulizations. To evaluate persistent pulmonary effects of a relatively brief EV aerosol exposure, animals were allowed a 10-day recovery period, with lung tissue harvested on day 15 (Figure 6A). Expectedly, at the time of harvest, EV-exposed and recovered hamsters showed no clinical signs of illness, and no body weight changes (Supplemental Figure 2A), confirming that the model was nontoxic. Histopathology showed no signs of inflammation or fibrosis (Supplemental Figure 2, B and C). EV-exposed animals showed a 4-fold increase in p62 abundance in the distal lung parenchyma, an increase in p62 abundance colocalizing with CD31-positive endothelial cells lining small pulmonary arteries, and a significant increase in p62 in epithelial cells lining small airways (Figure 6, B and C), consistent with impaired autophagic flux. We noted cleaved caspase-3 presence in EV-exposed lungs, even at 10 days after exposure (Figure 6D). Costaining for CD31 and terminal deoxynucleotidyl transferase–mediated dUTP nick end labeling (TUNEL) revealed that endothelial cells had DNA strand breaks at 10 days after EV exposure (Figure 6E). These results suggest modest but persistent apoptosis in distal lungs following EV exposure.

### Persistent transcriptomic responses in hamster lungs following brief EV exposures.

To comprehensively profile biological processes, signaling, and disease associations driven by EV exposure, we performed RNA-seq of hamster lungs and analyzed pathway enrichment. RNA-seq, filtered for log_2_ fold change greater than 1 or less than –1 and *P* less than 0.05, revealed about 200 upregulated and about 300 downregulated lung genes. In this analysis, while many genes achieved *P* value significance, far fewer met the more stringent FDR (*q*) threshold, likely because of brief, nontoxic exposure followed by recovery, the inherent variability in transcriptomic data, and the relatively low number of animals per group. However, the observed nominal significance of differentially expressed genes may provide biologically meaningful information that will require further corroboration.

**Figure 7:**
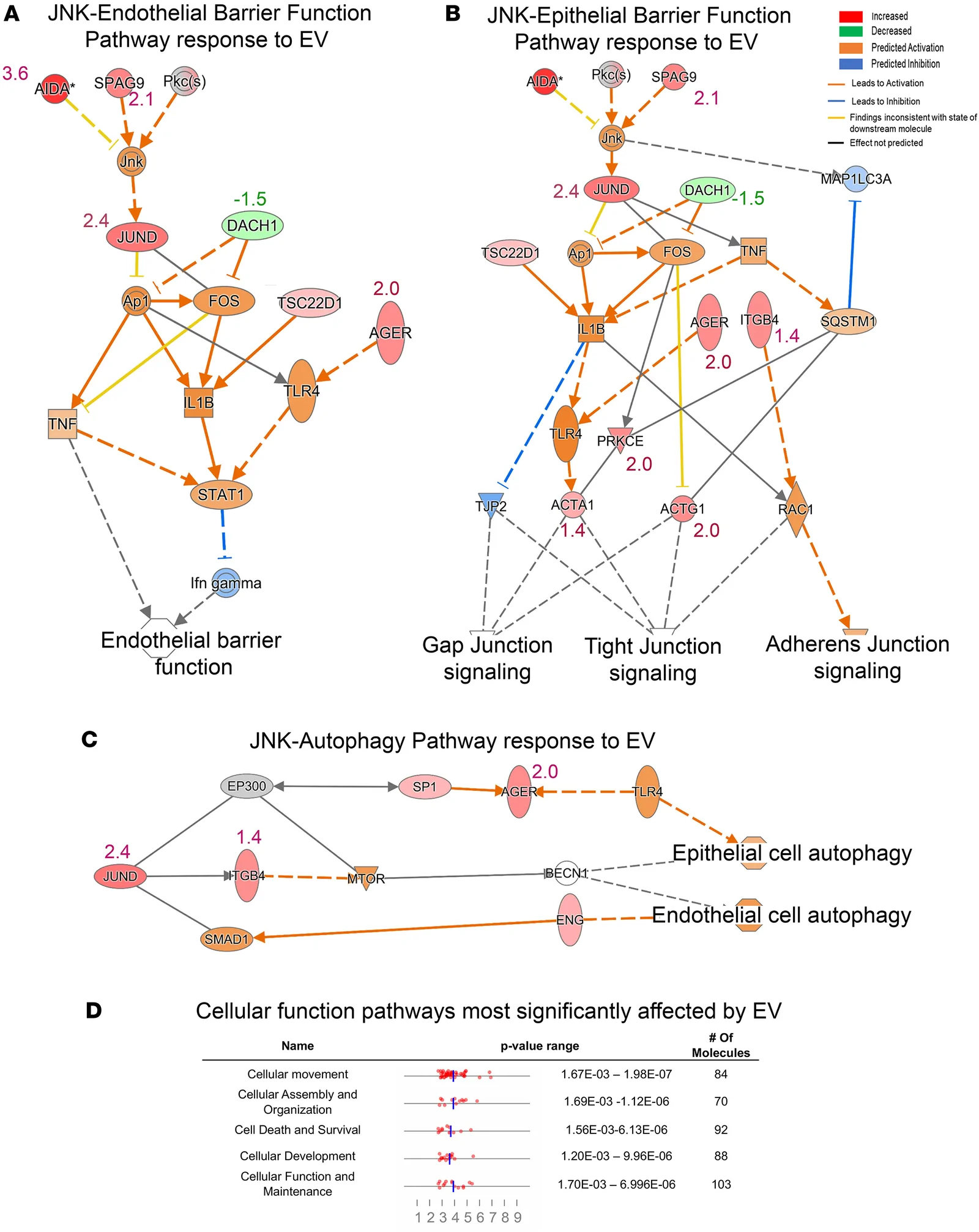
Interactivity map of JNK signaling induced by brief distant EV exposure in hamster lungs. (**A**–**C**) Persistent EV effects on JNK-anchored signaling pathways linked to endothelial barrier function (**A**), epithelial barrier function (**B**), and cell autophagy (**C**). Molecules that are predicted or measured as activated are shown in orange or red, respectively (with indicated log_2_ fold changes), and those that are predicted or measured to be inhibited are shown in blue or green, respectively. Note predicted JNK activation by upstream regulators (e.g., SPAG9, Pkcs, AIDA), and downstream activation of JunD and Ap1 and of proinflammatory mediators such as IL1B. Jnk is predicted to drive autophagy in epithelial and endothelial cells through pathways involving TLR4 and ENG, respectively. JunD activation (red), which promotes autophagy via transcriptional activation of ITGB4 and AGER, should lead to mTOR upregulation and subsequent autophagy regulation through BECN1. However, downstream mTOR targets are not increased, suggesting its activity is suppressed. AGER/TLR4 signaling drives epithelial autophagy, whereas ENG signaling mediates endothelial autophagy. (**D**) Dot plot graph and summary table showing the *P* value distribution for pathways involved in cellular and molecular function and the corresponding number of molecules involved.

To validate the results obtained in human cells, we first interrogated the JNK signaling pathway. Although this was not the top differentially regulated pathway during the recovery from acute EV exposure, interactive pathway analysis predicted that EV activates the JNK signaling pathway as a result of (nominally) significant increase in expression of the upstream JNK activators *Spag9* (JNK-interacting protein-4; 4-fold; *P* = 0.03) and *Aida* (axin-interacting protein-1; 9-fold; *P* = 0.02) (Figure 7, A–C), as well as the downstream JNK mediator *JunD*, which was also increased 5-fold (*P* = 0.003). In addition to JunD, a central signaling pathway hub linking cellular stress to barrier function and autophagy, another notable effector change was the decrease in *Dach1* (Dachshund homolog 1), predicted to impair autophagy completion (27) and trigger lung epithelial cell apoptosis, promoting fibrosis (28). EV decreased *Dach1* by 50% (*P* = 0.0007) while upregulating *Ager* (advanced glycation end product receptor), *Eng* (endoglin), *IL1β*, *Itgb4* (integrin β_4_), and *Tlr4*, which may link EV-induced JNK activation to barrier dysfunction and autophagy. These results demonstrate cross-validation between the human cell experiments and the animal model, reinforcing the biological significance of the identified pathways.

We determined which transcripts are most differentially modified by remote EV exposure in hamster lungs. The top 20 transcripts that were most increased by prior EV exposure in hamster lungs and that reached nominal statistical significance (Table 1) included genes involved in protein degradation (such as ~40-fold increase in ubiquitin-conjugating enzyme E2 O), innate immune defense (~14-fold increase in BPI fold containing family B member 1), coagulation and inflammation (such as ~13-fold increase in tissue factor), cell adhesion and proliferation (such as tumor-associated calcium signal transducer 2 and LY6/PLAUR domain containing 2), and stress response (Sumo1/sentrin peptidase 1, Nudix hydrolase 13, and JunD). The 20 transcripts most decreased by prior EV exposure in hamster lungs and that reached nominal statistical significance (Table 2) were also genes involved primarily in proteostasis and matrix remodeling, such as ubiquitin-conjugating enzyme E2 D1, protease, serine 35, and osteopontin, with *Ubl5* (ubiquitin-like protein 5) registering a statistically significant decrease of more than 600-fold (*P* = 4.51 × 10^–5^, *q* = 0.04). Also downregulated were genes involved in mitochondrial function and the immune response, such as galectin-9, Cxcr2, and Nod2. These findings suggest that EV contributes to a persistent stress response and signaling that impact immune function and tissue remodeling.

When examined by enrichment scores, the top 5 functional categories of gene pathways impacted by EV (Figure 7D) included cellular movement, followed by the cellular assembly and organization pathway, suggesting cytoskeletal remodeling; cell death and survival; cellular development; and cellular function and maintenance. These pathways are consistent with EV-triggered activation of autophagy, stress-adaptive survival, and cellular repair mechanisms.

When analyzed using patterns related to biological responses or disease associations, pathway enrichment analysis revealed significant activation of actin cytoskeleton, hepatic fibrosis, and neutrophil extracellular trap signaling (Supplemental Figure 3A) as well as cancer, inflammatory disease, and cell-mediated immune response (Supplemental Figure 3B). Interestingly, gene expression was predominantly inhibited in NET, RHOGDI, and Th1 pathways, suggesting immune suppression or impaired inflammatory resolution. Other activated pathways were linked to metabolism and endocrine regulation, with notable enrichment of the P2Y purinergic receptor signaling pathway, given its key role in airway inflammation, mucus hypersecretion, vascular reactivity, and pulmonary surfactant homeostasis.

### Short-term EV exposure alters host lung responses to acute respiratory viral infections in hamster lungs.

**Figure 8:**
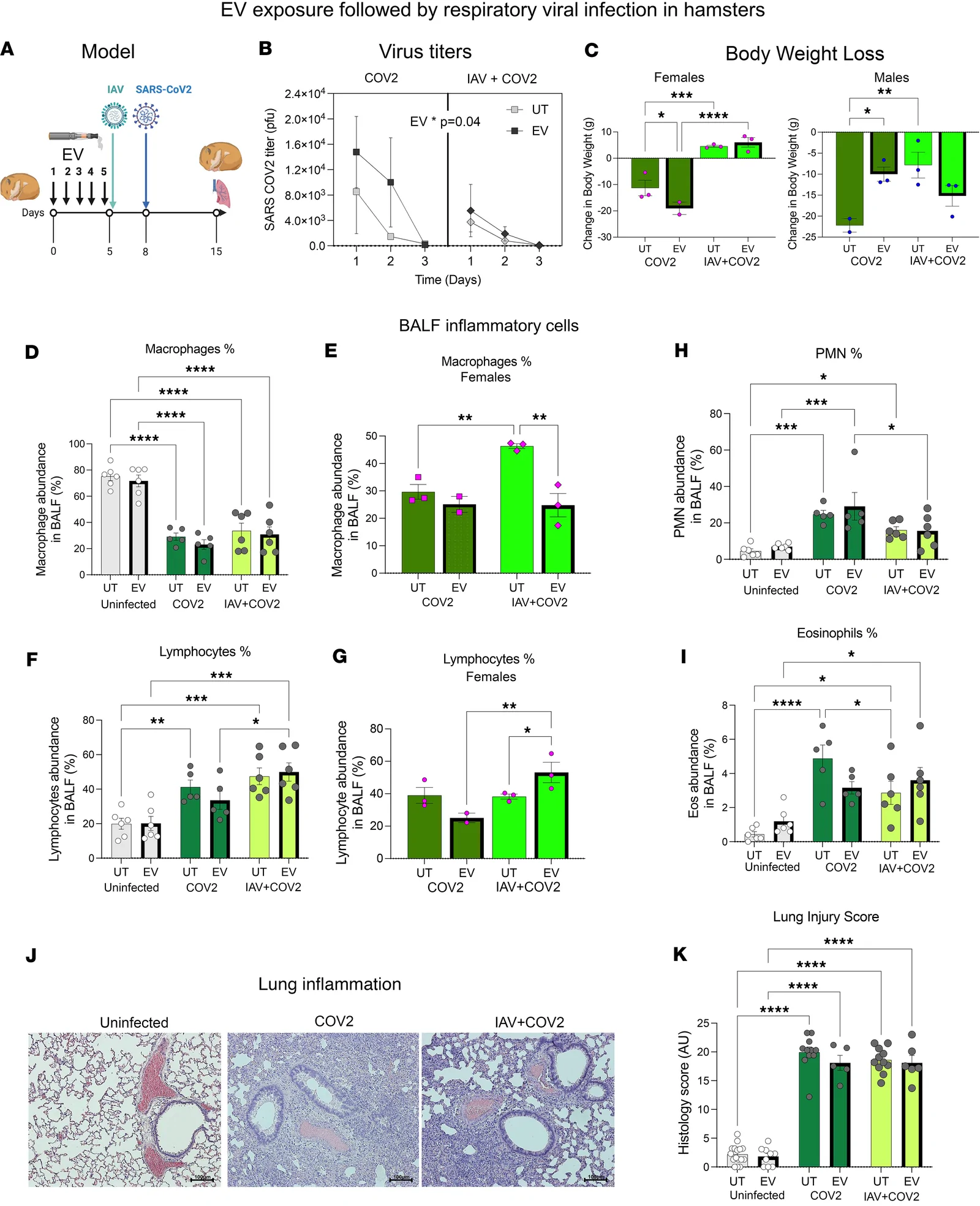
Effects of brief prior EV exposure on inflammatory responses to respiratory viral infections in hamsters. (**A**) Experimental timeline: Exposure to EV for 5 days prior to intranasal administration of influenza A virus (IAV), followed 3 days later by intranasal administration of SARS-CoV-2 (COV2). Viral titers were measured at days 1, 2, and 3 after infection with SARS-CoV-2; lung tissues and bronchoalveolar lavage fluid (BALF) were collected at 7 days after infection with SARS-CoV-2 (10 days after cessation of EV exposure). (**B**) Upper respiratory viral titers obtained by nasal swabs at the indicated times (days) after SARS-CoV-2 infection. Mean ± SEM; 3-way ANOVA with a *P* value for the EV of 0.04. (**C**) Change in body weight in female and male hamsters from pre-infection weight. Mean ± SEM; 2-way ANOVA with Fisher’s least significant difference (LSD). (**D**–**I**) Quantification of indicated inflammatory cell population abundance in cytospins of BALF. Mean ± SEM; 2-way ANOVA with Fisher’s LSD. (**J**) Representative H&E-stained lung tissue sections from uninfected, SARS-CoV-2–infected, and coinfected (IAV+COV-2) hamsters show exuberant inflammatory infiltrate in SARS-CoV-2–infected lungs. Scale bars: 100 μm. (**K**) Unbiased histological scoring of inflammatory lung injury (based on epithelial thickening and inflammatory cell infiltration). Mean ± SEM; 2-way ANOVA with Fisher’s LSD. For all graphs, *P < 0.05, **P < 0.01, **P < 0.001, ****P < 0.0001; dots are individual animals. UT, untreated.

The transcriptional changes in immune response pathways indicate that EV may modulate host responses to respiratory viral infections. We designed the experiments to address the following two questions: does EV pre-exposure modify the lung injury response to SARS-CoV-2; and does EV pre-exposure modify the effect of co-viral infection (with IAV) on the lung injury induced by SARS-CoV-2? The goal was not to evaluate (the effect of EV on) IAV-induced acute lung injury; rather, IAV served solely as a coinfection with SARS-CoV-2, reflecting a common clinical scenario. Given our focus on SARS-CoV-2–mediated injury, we selected a literature-supported time point corresponding to peak SARS-CoV-2 lung injury, and we exposed hamsters to daily EV for 5 days, followed by SARS-COV-2 (3 days later); or, sequentially, to both IAV (the following day) and SARS-COV-2 (3 days later) (Figure 8A). Lungs were harvested on day 7 after SARS-CoV-2 infection (during the injury phase). This experimental design also enabled us to test the effect of IAV pre-infection on host responses to SARS-CoV-2, with or without EV exposure.

To test the effect of EV on upper airway virus infectivity, we measured oropharyngeal SARS-COV-2 viral titers during the first 3 days following infection (Figure 8B). A 3-way ANOVA factoring time, EV exposure, and IAV infection showed that time (*P* = 0.004) played a significant role, and that EV significantly increased viral titers of SARS-COV-2 (*P* = 0.04) (Figure 8B). Although IAV infection reduced SARS-COV-2 titers, this did not reach statistical significance in this analysis (*P* = 0.13). As expected, at 7 days after SARS-COV-2, animals had significant weight loss that was more pronounced in male animals (Supplemental Figure 2B). EV pre-exposure significantly (*P* = 0.04) worsened weight loss in female animals infected with SARS-COV-2 (Figure 8C), while males pre-exposed to EV had less weight loss (*P* < 0.0001) (Figure 8C). A 3-way ANOVA factoring EV exposure, IAV coinfection, and sex showed that IAV and sex had a significant impact on SARS-COV-2–induced weight loss (*P* = 0.004), with IAV attenuating and male sex accentuating the SARS-COV-2–induced weight loss, and that EV significantly interacted with these 2 variables (*P* = 0.0006), modifying their effect on weight loss (Supplemental Figure 2B).

Analysis of bronchoalveolar lavage fluid inflammatory cells revealed that SARS-CoV-2 significantly decreased the abundance of macrophages and increased that of lymphocytes, neutrophils, and eosinophils (Figure 8, D–I). EV pre-exposure decreased macrophage (*P* = 0.01) and increased lymphocyte (*P* = 0.04) abundance only in female animals coinfected with IAV and SARS-CoV-2 (Figure 8, E and G). A 3-way ANOVA factoring EV exposure, sex, and IAV coinfection showed a significant effect of IAV on (increasing) lymphocyte predominance and (decreasing) neutrophil predominance in response to SARS-COV-2 (*P* < 0.05 for each) (Supplemental Figure 2, D and E), consistent with interference, and confirmed significant interaction of EV with sex and with sex and IAV on macrophage abundance (*P* < 0.05 for each) (Supplemental Figure 2C).

Histological analysis was consistent with increased inflammatory injury induced by SARS-CoV-2 in comparison with uninfected lungs, regardless of EV exposure (Figure 8, J and K). A 3-way ANOVA factoring EV exposure, sex, and IAV coinfection showed only a trend of impact of EV (*P* = 0.09) and sex (*P* = 0.07) on lung injury induced by SARS-CoV-2 (Supplemental Figure 2G).

Overall, these results show that remote short-term exposure to EV is sufficient to alter the susceptibility to respiratory infection with SARS-CoV-2, as evidenced by increased viral titers and (sex-dimorphic) weight loss. However, these changes were insufficient to alter lung inflammation and injury in response to SARS-CoV-2. Our data also indicate that 3 days of pre-infection with IAV was sufficient to interfere with SARS-CoV-2 infection, reducing its infectivity and its detrimental effect on weight loss, preventing the decrease in, and even increasing, macrophage abundance, increasing lymphocytes, and decreasing eosinophils. EV was synergistic with IAV in its effects on body weight and macrophage and lymphocyte inflammation in females, suggesting it may share mechanistic features of viral interference.

### EV exposure changes transcriptome responses to respiratory viral infections.

To gain insight into potential mechanisms underlying antiviral host responses triggered by EV, the transcriptomic profiles of lungs pre-exposed to EV and then infected with SARS-CoV-2 were compared with those of animals infected but unexposed to EV. Several significant effects were noted (Table 3), including EV increases in *Mmp13* (3-fold; *P* = 9.0 × 10^–7^, *q* = 0.001), *Mmp12* (3-fold; *P* = 0.0001, *q* = 0.035), and *osteopontin* (*Spp1*; 2-fold; *P* = 0.0002, *q* = 0.05) that, coupled with downregulation of *Timp1* and *Fmod*, suggested that EV enhances lung tissue remodeling following virus infection. Downregulation of *Slc31a1* is essential for cytochrome *c* oxidase (by 30%, *P* = 4.2 × 10^–14^, *q* = 3.87 × 10^–10^) and for critical components of the electron transport chain, indicating significant effects on mitochondrial responses to viral infection. EV-pre-exposed lungs responded to SARS-CoV-2 with significantly increased neuritin (*NRN1*; 4-fold; *P* = 9.26 × 10^–5^, *q* = 0.033), a pro-angiogenic molecule with neuroendocrine signaling effects, and decreased TMF-regulated nuclear protein 1 (*TRNP1*; by 80%; *P* = 2.7 × 10^–10^), an inhibitor of cell proliferation. Pathway analysis showed that lungs pre-exposed to EV exhibited decreased phagosome formation, decreased immune cell death, and enhanced NETosis, IL-12 signaling, muscle cell death, and vascular lesion formation in response to SARS-CoV-2 infection (Supplemental Figure 4A). When EV preceded both IAV and SARS-CoV-2, similar patterns of gene expression were observed, with EV-exposed lungs showing upregulation of genes critical for extracellular matrix integrity and downregulation of genes involved in energy production and protein synthesis (Table 4).

Pathway analyses indicated activation of fibrosis and actin cytoskeletal reorganization (Supplementary Figure 4A) and downregulation of antiviral genes and innate immune responses, as well as those involved in immune recruitment and resolution (Supplemental Figure 4B). Signaling by IL-12, a central Th1 immune cytokine that stimulates IFN production and IL-23 and suppresses Th17 cell responses, was increased by EV in doubly infected lungs (Supplemental Figure 4, C and D). These changes suggest that EV exposures are favorable for viral persistence and may promote chronic inflammation in response to viral infections.

IAV pre-infection altered transcriptional responses to SARS-COV-2 infection, enhancing pulmonary and hepatic fibrosis pathways (Supplemental Figure 5A), increasing estrogen receptor signaling (Supplemental Figure 5, A and D), and suppressing interferon signaling (Supplemental Figure 5, B and C) and cell proliferation while increasing DNA damage and inhibiting coronavirus pathogenesis pathways (Supplemental Figure 5B).

## Discussion

These results indicate that even short-term EV exposure has detrimental effects on lung health, with EVs potentially directly injuring distal lung cells, triggering a stress response that decreases the barrier function of small airway epithelial and lung microvascular endothelial cells, and leading to incomplete cell repair via stress-autophagy that may culminate in apoptosis. This stress response persists for at least 10 days after short-term EV exposures, with transcriptomic changes that predict decreased immune defenses against respiratory viruses, and notable alterations in proteostasis and tissue remodeling that might herald the development of chronic lung diseases following habitual EV use.

The pulmonary consequences of vaping remain an important public health concern marked by important knowledge gaps and the misconception that EV is a safer alternative to cigarette smoking. By focusing on the distal lung, our study addresses unmet needs (29), complementing knowledge of the harmful effects of EV on the proximal airways (30–32) or on specific cell populations (33). By using a popular commercial EV product and integrating studies of human distal lung structural cells with those in laboratory animals that share distal lung anatomy and immune responses to respiratory virus infection with humans, our study has clinical relevance. However, we did not dissect potentially distinct (11, 12, 34–36) nicotine-dependent versus nicotine-independent effects, or those of various flavorings, and, owing to BSL-3 regulations at the time of the study, we relied on aerosolizing EV from condensate, which lacks short-lived volatile compounds.

Acute EV exposure of human lung endothelial and epithelial cells disrupted the distal lung structural cell barrier function, thereby weakening lung defenses and promoting inflammation, a process pertinent to the development of several chronic lung diseases, including COPD and lung fibrosis, which are also characterized by decreased lung barrier function. Building on previous findings that EV decreases lung microvascular endothelial cell barrier function (11), current results add mechanistic insight and demonstrate a similar permeability increase in small airway epithelial cells when exposed to EV experimental formulation as vapor, juice, or condensate.

Both cell types exposed to EV underwent autophagy with incomplete autophagolysosomal degradation and inefficient repair, as indicated by increased apoptosis. There is ample evidence that stress-induced autophagy is coupled with decreased cell proliferation rates to enable cell survival and repair (37). However, autophagy can directly precede and trigger apoptosis if stress levels are excessive and persistent, or if autophagy is impaired and/or decoupled from other cell repair processes (38). Acute EV exposures induced enrichment of ceramide in the plasma membrane and nuclear translocation of caspase-3, events that signify apoptosis (39–44). Although the levels of EV exposure studied did not induce massive apoptosis, and apoptotic markers were recorded many hours after the loss of barrier function, it is possible that apoptosis could weaken the barrier of the microvascular endothelium and the small airway epithelium. Apoptosis was also modest during recovery from EV exposure in hamster lungs and was primarily found in the distal lungs and lung endothelial cells.

Our study implicated JNK as a key signaling event in both EV-induced barrier dysfunction and dysfunctional autophagy. Other MAPKs activated by stress, such as p38 MAPK (45), have been implicated in the effects of EV on neutrophils (46), but not on rat lung microvascular endothelial barrier dysfunction (11). In previous studies, we identified both p38 and JNK as mediators of cigarette smoke–induced cytoskeletal changes in lung endothelial cells, but not barrier dysfunction (47). Interestingly, JNK inhibition did not affect S6 phosphorylation, suggesting the presence of additional mechanisms of mTOR signaling during EV exposure.

As expected (31, 48), short-term EV exposures in hamsters, as in mice, and especially when followed by a recovery window, are insufficient to trigger appreciable systemic changes. Our data build on findings published by Hinds et al. (24) on 2-day whole-body nicotine solution exposure in hamsters, with immediate histological and targeted gene transcription assessments showing increases in the inflammation-related genes IL-1β, ELANE (NETosis gene), and tissue factor and in profibrotic genes. Our study complements these results by capturing previously unreported persistent effects of acute EV exposures and by providing a much broader transcriptomic assessment, which shows that IL-6 elevations (2.2-fold; *P* = 0.01) and IL-1β reductions (by 50%; *P* = 0.01) persist even after EV cessation. In addition, we show that EV induces persistent changes in cytoskeletal dynamics and cell death and survival pathways, suggesting that it amplifies inflammation, promotes tissue remodeling, and impairs immune function. Repeated activation of these pathways could lead to persistent airway and vascular remodeling observed in chronic lung diseases. Pathway analysis following sequential EV, IAV, and/or SARS-CoV-2 exposures suggests that EV has a detrimental effect on the lung’s ability to respond to SARS-CoV-2 infection, driven by a complex interplay between antiviral immune suppression, augmented tissue remodeling, and mitochondrial dysfunction signaling. Although not a study focus, nonlethal IAV pre-infection had an overall protective, antiinflammatory priming effect on subsequent SARS-CoV-2 infection, also known as interference (49). In contrast, EV exerted a detrimental priming effect on respiratory viral infections, with exaggerated inflammatory and tissue remodeling responses and impaired injury resolution. We noted a significant interaction between EV effects and animal sex. Given the small number of animals in each sex subgroup, these findings will need to be validated in future studies. Another limitation is that we did not investigate the effect of EVs on IAV-induced lung injury or recovery, as this topic has recently been explored. Instead, we used IAV to model a coinfection with SARS-CoV-2, a common clinical scenario (50, 51).

In conclusion, this study finds that even short-term EV exposure activates stress responses, induces cell injury, and reduces repair in human lung parenchyma cells, changes that persist after EV exposure. Even brief EV exposures decrease antiviral immunity, increasing susceptibility to SARS-CoV-2 and influenza virus infections while triggering molecular pathways involved in lung tissue remodeling. Identifying JNK signaling as a modifiable pathway may guide future research on therapeutic strategies to mitigate EV-induced lung injury. These findings provide mechanistic evidence linking EV exposure to impaired lung defense and distal lung remodeling, such as lung fibrosis, informing risk assessments of EV safety and prevention measures against respiratory virus exposures in habitual EV users.

## Methods

### Sex as a biological variable.

Both sexes were included in human-cell-based and animal studies. When available, both sexes were used equally in the study design and interpretation.

### Reagents.

All reagents were purchased from Sigma-Aldrich/EMD Millipore unless otherwise stated.

### Cell cultures.

All cell cultures were maintained in sterile conditions at 37°C and 5% CO_2_. Primary human lung microvascular endothelial cells (HLMVECs) were obtained from Lonza (CC-2527) and maintained in their appropriate media with standard growth factor supplements (Lonza). Immortalized HLMVECs (HULECs, ATCC, CRL-3244) were grown in MCDB131 (Corning, 45001-112) medium and used in submerged culture. h-TERT–immortalized human small airway epithelial cells (hSAEC1-KT, ATCC, CRL 4050) were grown in PneumaCult-Ex Plus Medium (StemCell Technologies, 05040), characterized for full differentiation at air-liquid interface (Supplemental Figure 6), and used in experiments at earlier stages of differentiation, while submerged in medium. Primary hSAECs were isolated from lungs of deidentified organ donors that had not been used for transplantation and that were donated for medical research. The lungs were obtained from the National Jewish Health Human Lung Tissue Core, which obtains deidentified lungs through the National Disease Research Interchange (Philadelphia, Pennsylvania, USA), the International Institute for the Advancement of Medicine (Edison, New Jersey, USA), or the Donor Alliance of Colorado (Denver, Colorado, USA). The Committee for the Protection of Human Subjects at National Jewish Health, Denver, Colorado, deemed this research as exempt from IRB human research. Donors were without a history of lung disease, were lifelong non-smokers, and had no lung injury as indicated by a PaO_2_/FiO_2_ greater than 300, a chest radiograph that indicated no acute process, and a time on the ventilator of less than 5 days. Small airway (≤2 mm diameter) epithelial cells were collected from the distal lung using a 2 mm bronchoscopy brush (Conmed) and placed in sterile PBS. Cells were isolated by centrifugation, resuspended in PBS, counted, and plated onto an irradiated NIH 3T3 fibroblast feeder layer in F-medium. Once visible colonies had formed (7–10 days), they were removed with 0.25% trypsin (Corning, 25-053-CI) and plated on double-collagen-coated 12-well Transwells (Advanced BioMatrix, 5005, and Corning, 3460). Cells were maintained in PneumaCult-ALI-S medium (StemCell Technologies, 05001) at 5% CO_2_ and 37°C for 21 days.

### Human PCLSs.

Right upper lung lobes from deidentified non-smoking donors with no lung disease or infection were obtained from the International Institute for the Advancement of Medicine or the Donor Alliance of Colorado. Lungs were inflated with 1.5% low-melting agarose (42°C) and sliced into consecutive sections of 450 μm thickness using a Compresstome VF-300 vibratome (Precisionary Instruments). The slices were transferred to 24-well plates containing Dulbecco’s modified Eagle medium (Thermo Fisher Scientific) with antifungal agents and antibiotics and incubated in a humidified incubator at 37°C, 5% CO_2_.

### EV exposure of cells.

To generate EV, we used JUULpods with 5% (59 mg/mL) nicotine, commercialized as Virginia Tobacco (JUUL), with a ratio of 30:70 propylene glycol/vegetable glycerin. We tested several forms of EV, as juice or aerosol, in submerged and air-liquid interface exposure models, respectively. For submerged cultures, to determine whether the vaporized juice has similar effects to the non-vaporized juice on the outcomes tested, we generated EV condensate.

JUUL juice was prepared by dilution of JUUL liquid in serum-free medium to a 10× stock solution that was then added to submerged cell cultures or PCLSs to achieve a final concentration of 2.5–10 mM nicotine. A VITROCELL VC 1 apparatus (VITROCELL Systems GmbH) was used to generate EV aerosol, which was used to either obtain EV condensate used in submerged cultures, or directly expose to EV SAECs at the air-liquid interface. The VITROCELL protocol consisted of a modified Cooperation CORESTA-recommended method 81, with puff volume, 55 mL; puff duration, 3 seconds; puff frequency, 30 seconds; puff hold time, 0 seconds; puff exhaust time, 8 seconds; and puff number, 75 puffs. The EV condensate collected was considered 100% and used at a 5% final concentration in submerged cultures, with ambient air used to generate a control condensate at similar concentrations. For experiments using inhibitors, cells or PCLSs were exposed to EV once at the indicated final nicotine concentrations in the presence or absence of pan-JNK inhibitor (SP600125; 50 μM, 10 mg; Selleckchem, S1460), ERK1/2 inhibitor (PD98059; 50 μM; Fisher Scientific, NC0771630), p38 MAPK inhibitor (SB202190; 30 μM, 5 mg; Sigma-Aldrich, S7067), or DMSO as vehicle control.

Cell monolayer barrier measurement was performed using ECIS (Applied Biophysics) with the Z-Theta 16-well array station. Cell monolayers were grown on 8W10E or 8W10E+ Cultureware to a capacitance of <1 nF or <10 nF, respectively, at 64 kHz. Measurements of electrical resistance (ohms) were collected at 4,000 Hz over time. A complementary assay to measure the barrier function of cells grown on inserts (0.4 μm × 6.5 mm) used fluorescently labeled dextran (FITC or rhodamine B, 4K) applied to the apical side, followed by kinetic measurement of fluorescence in the basal compartment at excitation and emission wavelengths (FITC, excitation 485, emission 538; rhodamine B, excitation 544, emission 590).

### Immunoblotting.

Cells were resuspended in standard RIPA buffer (R0278, lot SLBL7395V) on ice for 30 minutes, collected by centrifugation, and quantified using standard BCA. Proteins were resolved by 4%–20% gradient PAGE and transferred using a semi-dry transfer apparatus (Bio-Rad) onto Immobilon-P PVDF membranes (MilliporeSigma, IPVH00010). Membranes were blocked in Pierce Protein-Free T20 (TBS) Blocking Buffer (Thermo Fisher Scientific, 37571) and washed in TBS with 0.1% Tween 20. Antibodies for immunoblotting were p62 (Abnova, H00008878M01), LC3B-II (Sigma-Aldrich, L7543), phospho-S6 (Cell Signaling Technologies, 2211S), anti-vinculin (Abcam, AB130007), and anti–β-actin (Fisher Scientific, A5441) and were incubated overnight at 4°C. Anti-mouse (NA931V, lot 9715064) and anti-rabbit (NA9340V, lot 10997954) HRP-conjugated secondary antibodies were from GE Healthcare.

### Cell immunofluorescence microscopy.

Human lung endothelial cells or hSAECs were plated on chamber slides (Falcon, culture slides, 354108) coated with either 12.5 μg/mL fibronectin in PBS or 50 μg/mL collagen type I in 0.02N glacial acetic acid, respectively. Experiments were conducted on cell monolayers at about 90% confluence. At the end of experiments, cells were fixed in 4% paraformaldehyde in PBS for 20 minutes at room temperature, then permeabilized in 0.5% of Triton X-100 in PBS.

For cell proliferation measurements, cells were incubated with the fluorescently labeled synthetic nucleotide EdU (5-ethynyl-2′-deoxyuridine) dissolved in DMSO (Invitrogen, C10637A), which was added 4 hours before experiment completion; briefly, 100× EdU solution in PBS was added to each cell culture for a final EdU concentration of 10 μM. The nuclear uptake of EdU was detected using the Invitrogen Click-iT Plus EdU Cell Proliferation Kit for Imaging, Alexa Fluor 488 dye (Thermo Fisher Scientific, C10637).

For immunofluorescence, after fixation and permeabilization, plated cells were blocked with 5% goat serum and 0.1% Triton X-100 in PBS for 1 hour at room temperature, then incubated overnight at 4°C with primary antibodies, followed by washing and incubation for 4 hours with respective secondary antibodies. This was followed by washing and nuclear staining with DAPI (400 ng/mL in 0.5% bovine serum albumin [BSA] and 0.01% Triton X-100 in PBS) for 20 minutes at room temperature, washing, and mounting using ProLong Diamond Antifade mounting solution (Invitrogen, P36970). Antibodies were diluted in an antibody buffer consisting of 1% BSA and 0.1% Triton X-100 in PBS. Primary antibodies were against p62 [1:100; mouse anti-SQSTM1–IgG2a(κ), Abnova, H00008878], LC3B (1:100; rabbit anti-LC3B, Sigma-Aldrich, L7543), PCNA [1:200; mouse anti-PCNA–IgG2a(κ), Novus, NB500-106], ceramides (1:200; mouse anti-ceramide–IgM [MID 15B4], Enzo, ALX-804196-T050), LAMP1 (1:100; rabbit anti-LAMP1, D2D11-XP, Cell Signaling Technology, 9091), and cleaved caspase-3 (1:200; rabbit anti–cleaved caspase-3, Abcam, ab2302). Primary antibodies were against mouse IgG2a(κ) (1:500; Alexa Fluor 594 goat anti-mouse IgG [H+L], Invitrogen, A11032), mouse IgM (1:500; Alexa Fluor 488 goat anti-mouse IgM [μ chain], Invitrogen, A21042), and rabbit IgG (1:500, Alexa Fluor Plus 488 goat anti-rabbit IgG [H+L], Invitrogen, A32731; or 1:500, Alexa Fluor 647 goat anti-mouse IgG [H+L], Invitrogen, A21245).

### Animal studies.

Golden Syrian hamsters (*n* = 6 per group, 3 months of age, male and female; Charles River) were exposed to EV (JUUL condensate; 70 puffs/100 μL/dose in saline) or vehicle (saline control) via nebulization. Sex as a biological variable was considered in the design (both sexes were included equally) and data analysis and interpretation. EV condensate was collected from the aerosol of vaped JUUL in a side-arm Erlenmeyer flask and solubilized in saline as follows: 7 pods (each 700 μL) were vaporized, and the condensate was brought up to 2 mL. Each animal was nebulized with EV or vehicle once per day for 5 consecutive days, and lungs were harvested 10 days after the last exposure (schematic in Figure 6A). The administration of nebulized EV was performed using a nebulizer unit (2.5–4.0 VMD, Aerogen) fitted with a cone that allowed nasal inhalation of the aerosol (5 minutes) by each animal at a time, as we previously reported (11). To assess the effect of EV on respiratory virus infections, hamsters were infected at the end of EV exposure, on day 5, with influenza A virus (IAV; A/California/07/2009) by intranasal instillation of 6 log_10_ PFU; and/or, on day 8, with SARS-CoV-2 (WA01) by intranasal instillation of 4 log_10_ PFU (schematic in Figure 8A). As controls, uninfected animals were instilled intranasally with 100 μL of PBS or 100 μL of medium, respectively. Hamsters were sacrificed 7 days after SARS-CoV-2 infection. The left lobe was clamped, and the right lung was instilled with PBS (1 mL ×3) and lavaged, with each return collected separately as bronchoalveolar lavage fluid (BALF). The first return was used for cytospins. The right caudal lung lobe was excised and placed in Buffer RLT (RNA Lysis Tissue, QIAGEN) with freshly added β-mercaptoethanol. The left lung was inflated with ethanol (70%) and then immersed in formalin (10%), sectioned with a random uniform sampling method, and mounted on cassettes for paraffin embedding.

### Lung histochemistry and immunofluorescence.

Cytospins obtained from the BALF were fixed and stained with a modified Diff-Quik stain using Giemsa Stain (Sigma-Aldrich, GS1L-1L). Samples were fixed to slides using 100% methanol for 10 minutes at room temperature, and then Giemsa solution (in water) was added for 20 minutes at room temperature. After staining, rinsed slides were air-dried and mounted with Cytoseal 60 (Fisher Scientific, 23-244257). Images were taken at ×20 magnification. Five hundred cells were assessed for each sample (blinded to the identity of the groups), differentiating among macrophages, lymphocytes, neutrophils, and eosinophils by morphological aspect and expressing their abundance as percentage of all cells counted.

For histological assessment, paraffin-embedded lung sections (4 μm thickness) were stained with hematoxylin and eosin (H&E) and then were assessed unbiasedly (blinded to the animal group identity) under light microscopy using a scoring system for inflammation as originally described (52) and previously applied for models of IAV lung infections (53) and hamster lung inflammation (54). The scoring was applied for each tissue section evaluated, using 5 criteria: (a) the number of bronchioles and bronchi with inflammatory cell infiltrate; (b) the severity of infiltrate of inflammatory cells in bronchioles and bronchi; (c) the severity of bronchiolar and bronchial luminal exudate; (d) the frequency of blood vessels with perivascular inflammatory cell infiltrate; and (e) the severity of pneumonia. H&E-stained slides were also assessed for lung fibrosis using the Ashcroft method, where each microscopic field of the biopsy was assigned an integer score from 0 (normal) to 8 (severe fibrosis).

For TUNEL staining, sections were deparaffinized in xylene, permeabilized with proteinase K, and postfixed with 4% paraformaldehyde. They were then washed in PBS, incubated in TdT buffer, and then washed with 0.1% Triton X-100 in 3% PBS/BSA before being incubated in TUNEL reaction solution. Samples were washed with PBS before blocking in PBS/BSA-T. The primary antibody (diluted in PBS/BSA) was added and incubated overnight at 4°C. Samples were washed twice before addition of the secondary antibody for 4 hours at room temperature. The samples were washed twice with PBS before being incubated with DAPI and mounted. Images were obtained on an Echo Revolution Microscope (Discover Echo Inc.).

### Bulk RNA-seq analyses.

Total RNA was isolated using an All Prep DNA/RNA/Protein kit (QIAGEN, 80004). Bulk RNA (RNA-seq) was performed by Novogene using Illumina RNA-seq technology. The sequencing data were mapped through STAR software (https://github.com/alexdobin/STAR/releases), replicate multivariate analysis of transcript splicing (rMATS) (https://rnaseq-mats.sourceforge.io/), and clusterProfiler (https://bioconductor.org/packages/release/bioc/html/clusterProfiler.html). The list of differentially expressed genes was analyzed with QIAGEN’s Ingenuity Pathway Analysis (IPA) using Core Analysis (datasets had a log_2_ fold change cutoff of ≥ ±1 and a *P* value cutoff of >0.05) to cross-reference against GO enrichment and Kyoto Encyclopedia of Genes and Genomes (KEGG) analysis results.

### Statistics.

Analyses of significance were performed using GraphPad Prism 10 software. A 2-tailed unpaired Student’s *t* test was used for experiments in which 2 conditions were being compared. A *P* value less than 0.05 was considered statistically significant. Comparisons between groups used ANOVA followed by an intergroup comparison with Tukey’s post hoc testing.

For RNA-seq analyses, a normalization method of DESeq2 was used, with the fold change being calculated with the equation [log_2_(*q_ij_*) = ∑*_r_* *X_jr_β_ir_*]. The *P* value was calculated using negative binomial distribution [*P*(*X* – *x*) = (*n* = *x* – 1 *n* – 1)*pn* (1 – *p*)*x*], and FDR was calculated using the Benjamini-Hochberg procedure: 
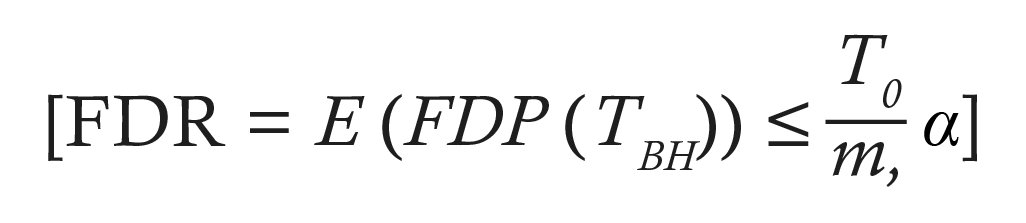


Differential gene screening was performed with thresholds of |log_2_(fold change)| ≥ 1 and adjusted *P* value ≤ 0.05. *Z* score was calculated through IPA software using 
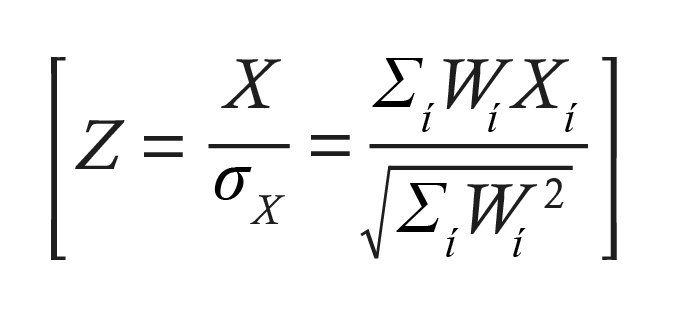
 Pathways with a *z* score ≥ ±2 were considered significant.

### Study approval.

The studies were performed in line with the principles of the Declaration of Helsinki. This study used human biological samples from deidentified individuals and was deemed IRB exempt. All studies were conducted under approvals by the institutional IACUC (no. 1372) at Colorado State University, Fort Collins, Colorado, USA and National Jewish Health, Denver, Colorado, USA and the Institutional Biosafety Committee at National Jewish Health (20-029B) protocols.

### Data availability.

Bulk RNA-seq data generated in this study are available in the Zenodo repository at https://doi.org/10.5281/zenodo.21676278 Note that the original FASTQ and BAM sequencing files are unavailable for public deposition because the archived files were corrupted and could not be recovered. Supporting data values associated with the main and supplemental figures are provided in the Supporting Data Values file.

## Author contributions

IP, HWC, and RB performed conceptualization. KSS, RB, EKV, JPB, SDG, GPD, and HWC developed methodology. TCR, KSS, IP, JPB, EKV, SDG, RAC, CM, CC, and JN performed investigation. NE performed visualization. IP and HWC acquired funding. IP and RB performed project administration. IP, RB, and HWC supervised the study. IP, TCR, and KSS wrote the original draft of the manuscript. IP reviewed and edited the manuscript. TCR assumed leadership of methodology and investigation after KSS relocated. TCR performed all pathway analyses and conducted experiments in SAECs; KSS oversaw the operationalization of the hamster experiment and coordinated with RB and the company performing RNA-seq, which conducted experiments in HLMVECs. They both contributed to writing. Therefore, they share first authorship.

## Conflict of interest

IP is a scientific co-founder and consultant with Allinaire Therapeutics.

## Funding support

This work is the result of NIH funding, in whole or in part, and is subject to the NIH Public Access Policy. Through acceptance of this federal funding, the NIH has been given the right to make the work publicly available in PubMed Central.

- American Lung Association grant ALA ETRA736704 (to IP).
- National Heart, Lung, and Blood Institute grants R01HL144396 (to IP and HWC) and R01HL077328 (to IP).
- Wollowick Chair for COPD Research at National Jewish Health (to IP).

## Supplementary Material

Supplemental data

Unedited blot and gel images

Supporting data values

## Figures and Tables

**Table 1:**
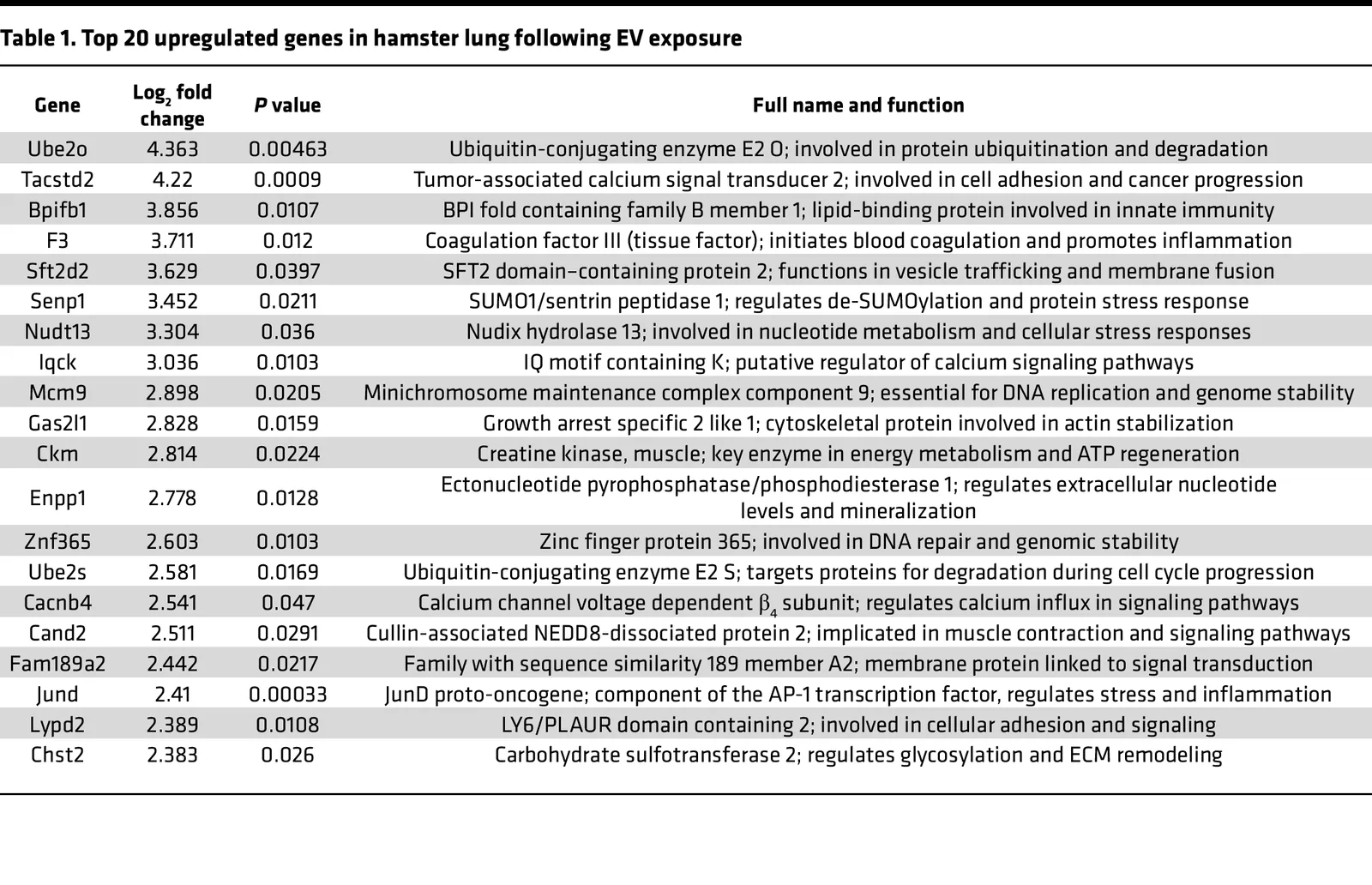
Top 20 upregulated genes in hamster lung following EV exposure

**Table 2:**
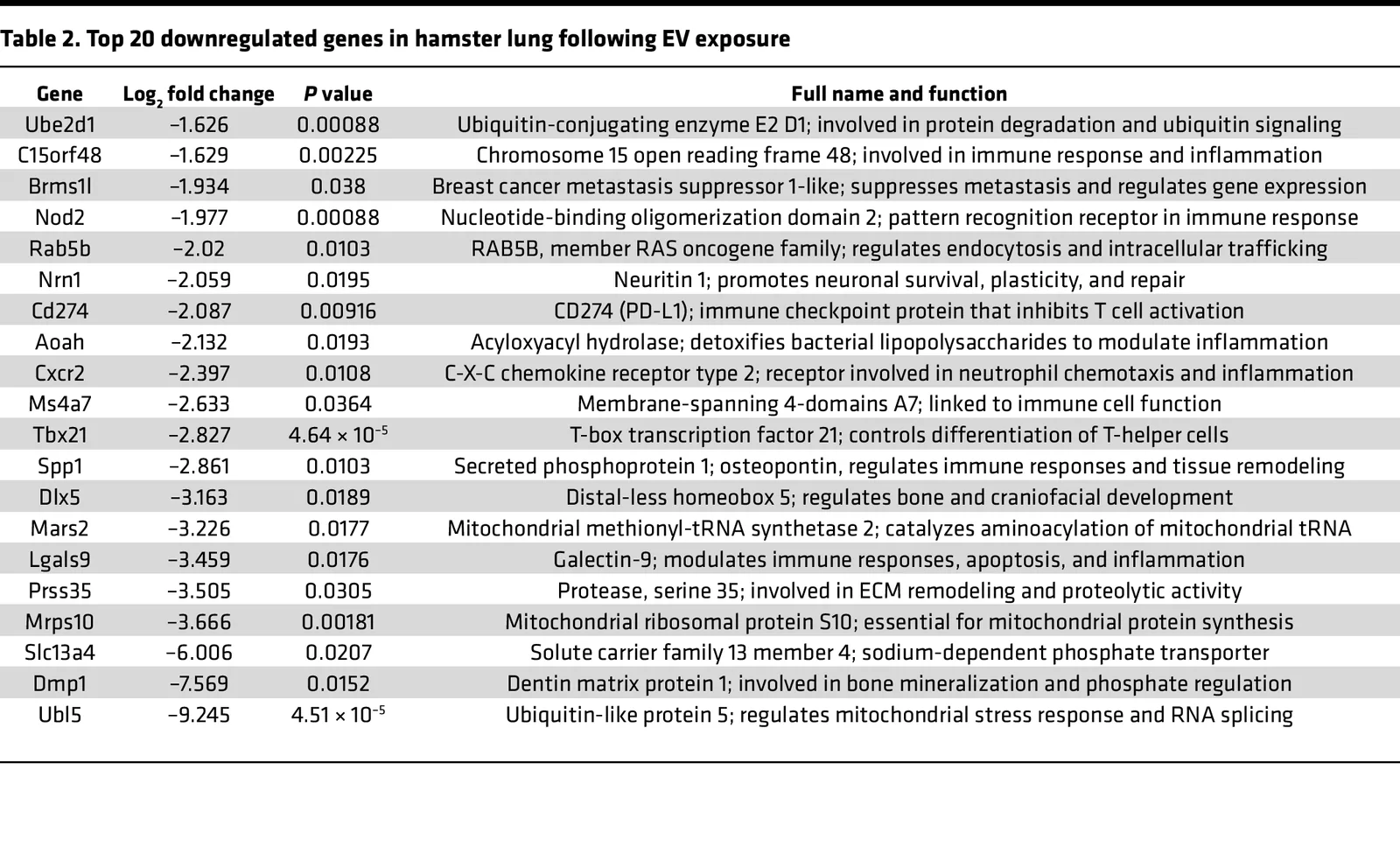
Top 20 downregulated genes in hamster lung following EV exposure

**Table 3:**
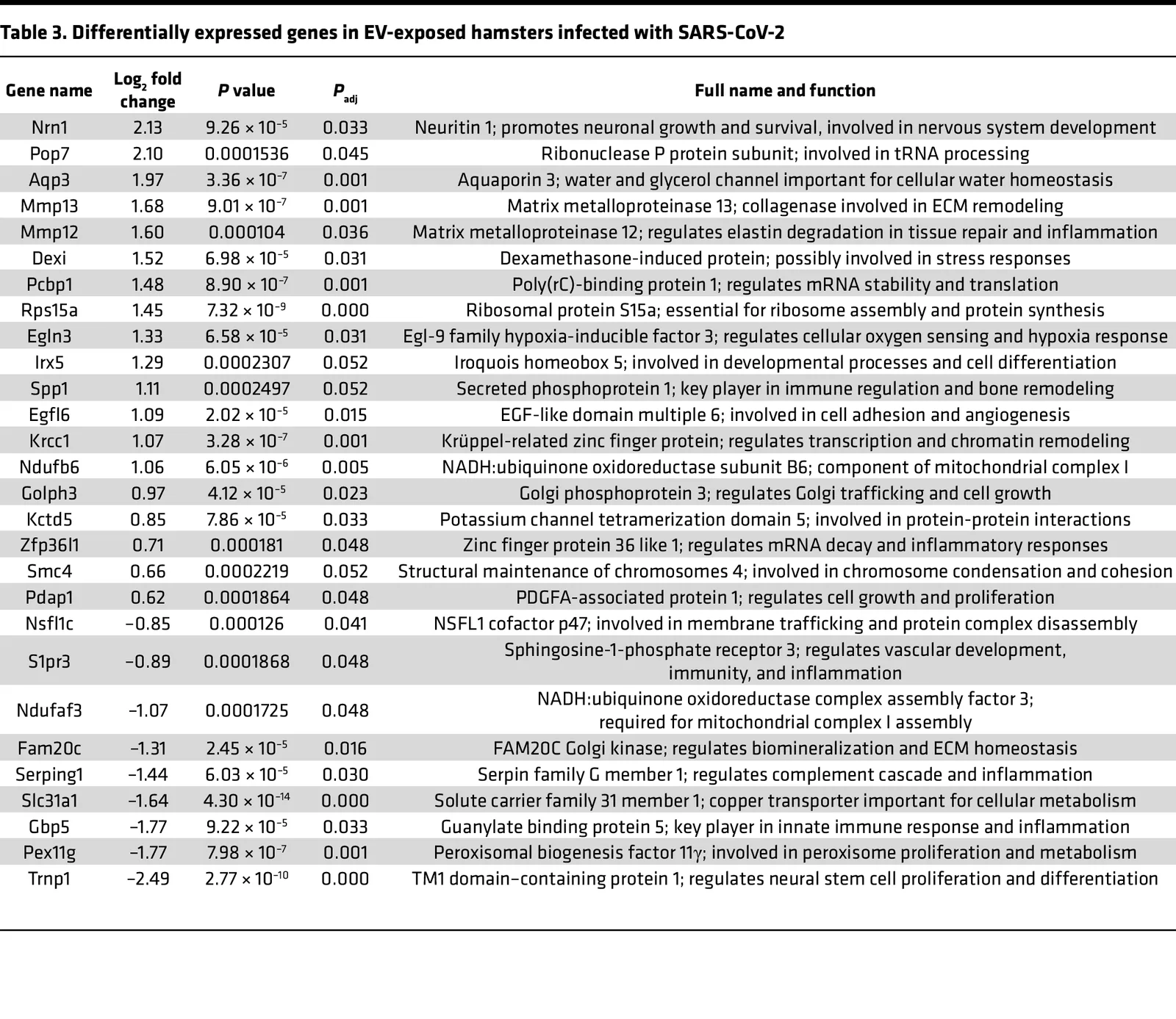
Differentially expressed genes in EV-exposed hamsters infected with SARS-CoV-2

**Table 4:**
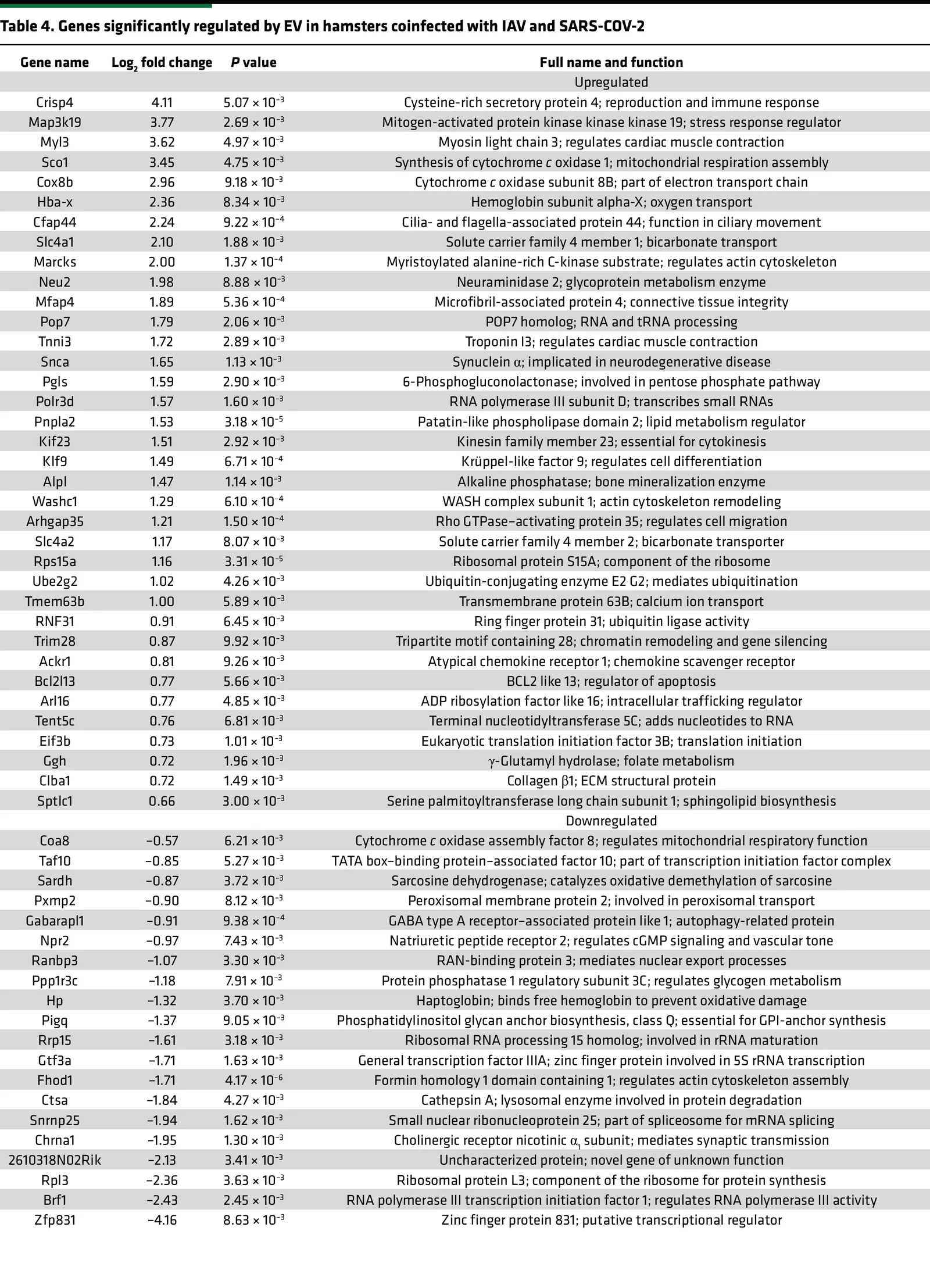
Genes significantly regulated by EV in hamsters coinfected with IAV and SARS-COV-2

## Version 1 08/11/2026

In-Press Preview

## Version 2 09/22/2026

Electronic publication
