# Supplementary material for: E-cigarette exposure triggers distal lung cell injury, persistent lung stress response, and antiviral immune suppression
Source: JCI Insight. 2026 Aug 11;11(18):e198757. doi: 10.1172/jci.insight.198757 (PMC13596730; doi:10.1172/jci.insight.198757)

Supplementary Figure 1.
Representative Immunofluorescence images of: **A**. Endothelial cells (HULEC) exposed to EV (7.5 mM; 24 hours) immunostained for LC3B (autophagy marker, green), LAMP1 (lysosomal marker, red), and DAPI (nuclear marker, blue). Note punctate staining for LC3B (arrow). **B**. Epithelial cells (hSAEC1-KT) exposed to EV (7.5 mM; 24 hours) immunostained for LC3B (autophagy marker, green), p62/SQSTM1 (autophagosomal marker, red), and DAPI (blue). Note punctate staining for p62 (yellow arrow), vacuole accumulation (white arrow), and varied distribution of LC3B, from perinuclear (pink arrow) to punctate colocalizing with p62 (blue arrow). UT = untreated.

Supplementary Figure 1

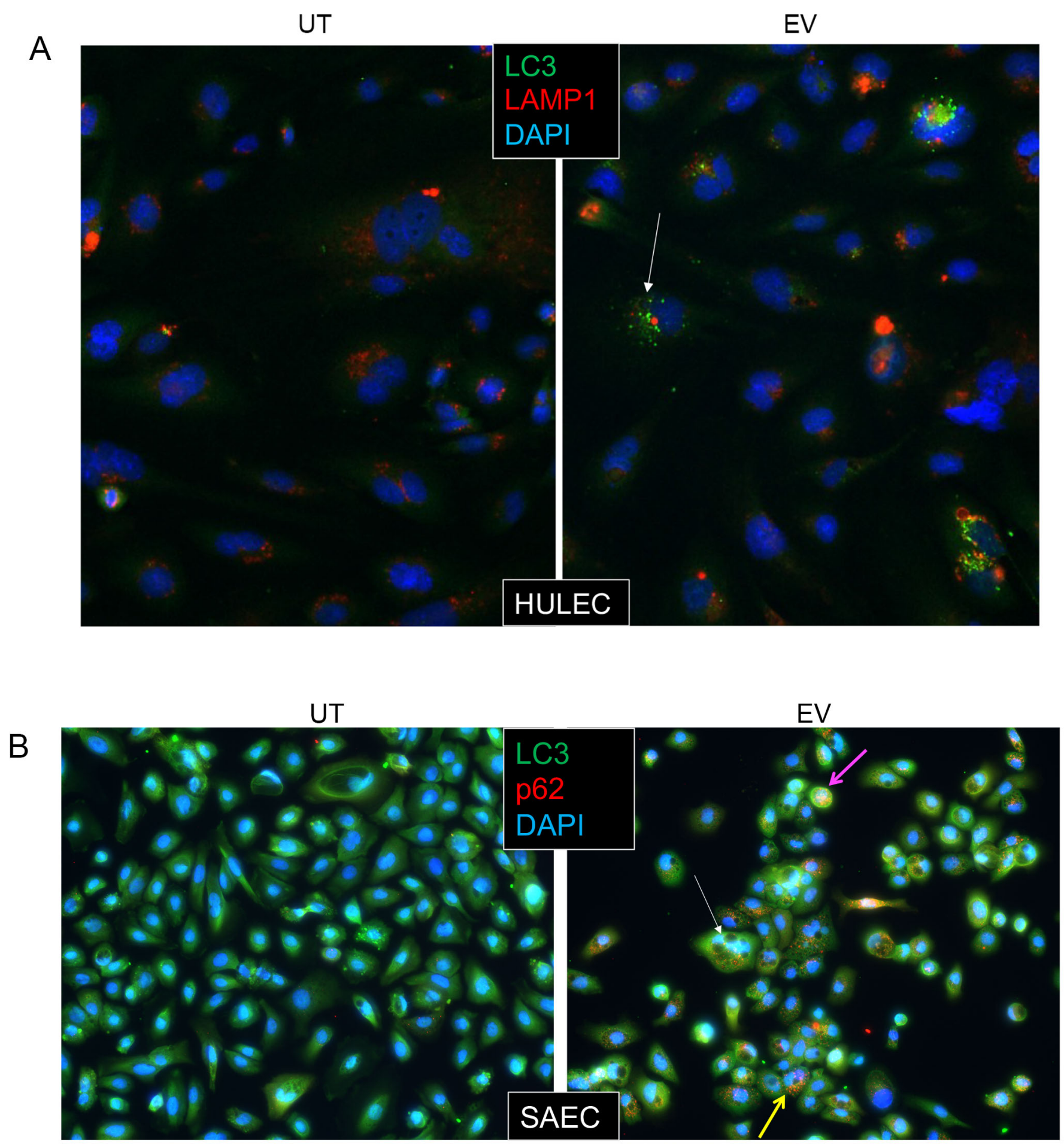

Supplementary Figure 2.

A. Representative H/E of the lung tissues in hamsters. Note the lack of appreciable changes in the lungs of animals previously exposed to a short-term EV inhalation followed by 10 days of recovery. **B**. Scatter plot with change in weight from the beginning of EV exposure to the time of harvest, at day 15, analyzed by sex. **C-F**. Scatter plot of inflammatory cell abundance (percent of total cells) in cytospins of BALF of hamsters exposed as indicated and analyzed by sex. **D**. Scatter plot of histology injury scores (arbitrary units) of hamster lung tissues. Tables below indicate 3 -way ANOVA analyses results, indicating the independent effect of EV, IAV, sex, or COV2, and any effect of their interactions. NS = not significant. UT = untreated.

Supplementary Figure 2

A

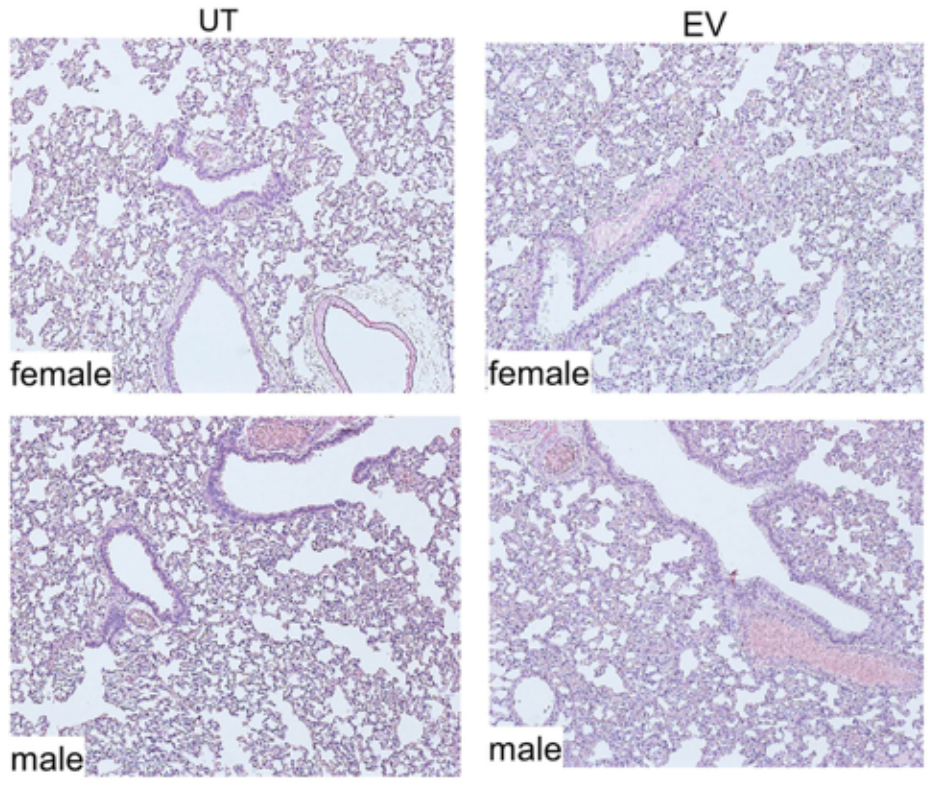

B

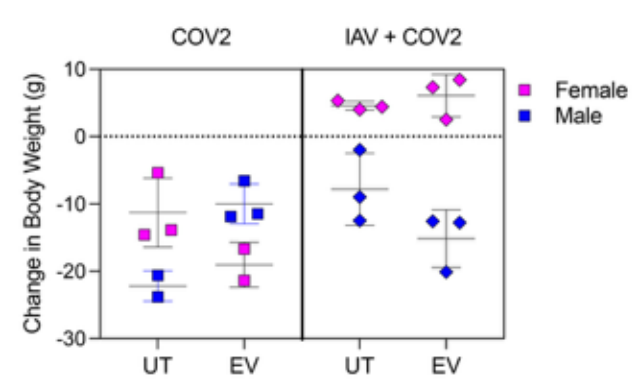

Source of Variation	% of total variation	P value	P value summary
EV	0.03021	0.8358	ns
IAV	39.76	<0.0001	****
Sex	19.88	<0.0001	****
EV x IAV	1.669	0.1388	ns
EV x Sex	1.951	0.1118	ns
IAV x Sex	15.95	0.0003	***
EV x IAV x Sex	13.08	0.0006	***

C

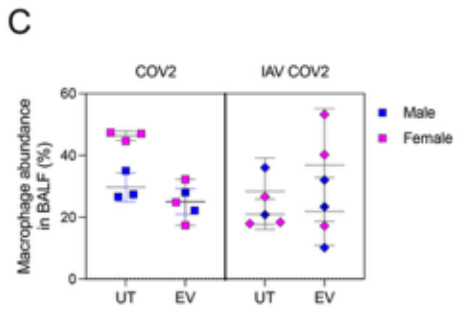

Source of Variation	% of total variation	P value	P value summary
EV	3.390	0.3223	ns
Sex	3.828	0.2940	ns
IAV	6.934	0.1644	ns
EV x Sex	15.21	0.0475	*
EV x IAV	0.3602	0.7430	ns
Sex x IAV	0.9346	0.5986	ns
EV x Sex x IAV	18.77	0.0301	*

3-way ANOVA
effects and interactions

D

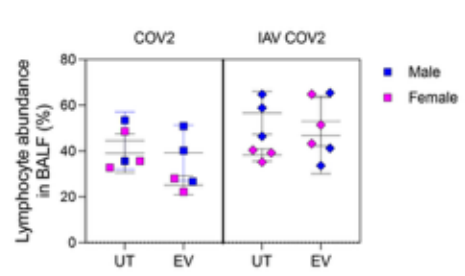

Source of Variation	% of total variation	P value	P value summary
EV	2.052	0.4532	ns
IAV	22.32	0.0234	*
Sex	10.08	0.1093	ns
EV x IAV	5.898	0.2119	ns
EV x Sex	2.647	0.3957	ns
IAV x Sex	0.5870	0.6861	ns
EV x IAV x Sex	11.27	0.0922	ns

3-way ANOVA
effects and interactions

E

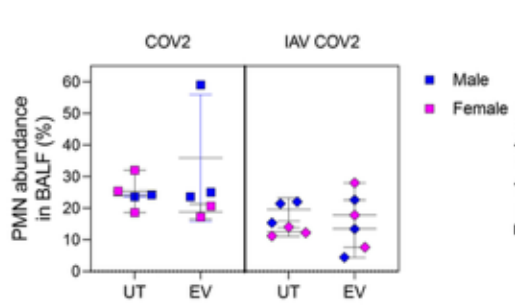

Source of Variation	% of total variation	P value	P value summary
EV	0.3044	0.7803	ns
IAV	22.30	0.0289	*
Sex	4.518	0.2918	ns
EV x IAV	0.5368	0.7114	ns
EV x Sex	0.6514	0.6838	ns
IAV x Sex	2.191	0.4582	ns
EV x IAV x Sex	12.00	0.0959	ns

3-way ANOVA
effects and interactions

F

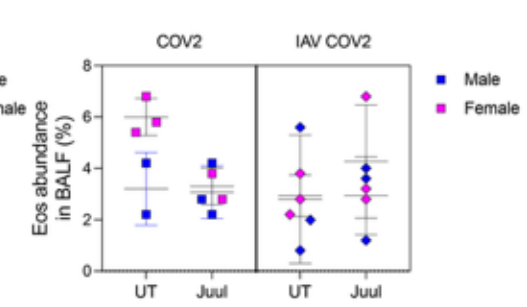

Source of Variation	% of total variation	P value	P value summary
EV	1.064	0.6199	ns
IAV	3.973	0.3436	ns
Sex	11.59	0.1162	ns
EV x IAV	10.58	0.1319	ns
EV x Sex	1.072	0.6185	ns
IAV x Sex	1.399	0.5700	ns
EV x IAV x Sex	8.100	0.1833	ns

3-way ANOVA
effects and interactions

G

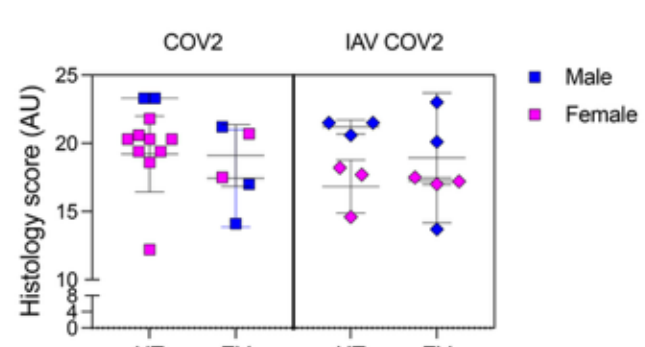

Source of Variation	% of total variation	P value
EV	9.687	0.0968
IAV	3.694	0.2947
Sex	11.34	0.0740
EV x IAV	2.661	0.3721
EV x Sex	11.17	0.0761
IAV x Sex	2.091	0.4278
EV x IAV x Sex	1.502	0.5006

3-way ANOVA
effects and interactions

Supplementary Figure 3.
Persistent effect of brief EV exposure on gene expression pathways in hamster lung. A-B. Pathway enrichment analysis of bulk RNA sequencing of lung tissue from EV-exposed hamsters**.** **A**. Bar graph of the top 20 enriched pathways based on -log(p-value) scores. Note pathways involved in cytoskeletal remodeling: Actin Cytoskeleton Signaling, ABRA (Actin-Binding Rho-Activating Protein) pathway, and the RHOGDI signaling pathways; inflammation: Neutrophil Extracellular Trap Signaling, Th1 Pathway; and metabolic regulation: GPCR-Mediated Nutrient Sensing, Insulin Secretion Signaling, Estrogen Receptor Signaling; suggesting a persistent (up to 10 days) integrated biological response to a relatively short (5 days) EV exposure. **B**. Bar graph with top-ranked pathways based on their statistical significance -log(p-value) categorized into disease-related and cellular functional processes, with higher -log(p-value) values indicating stronger enrichment. The yellow threshold line indicates the cutoff for statistical significance.

Supplementary Figure 3

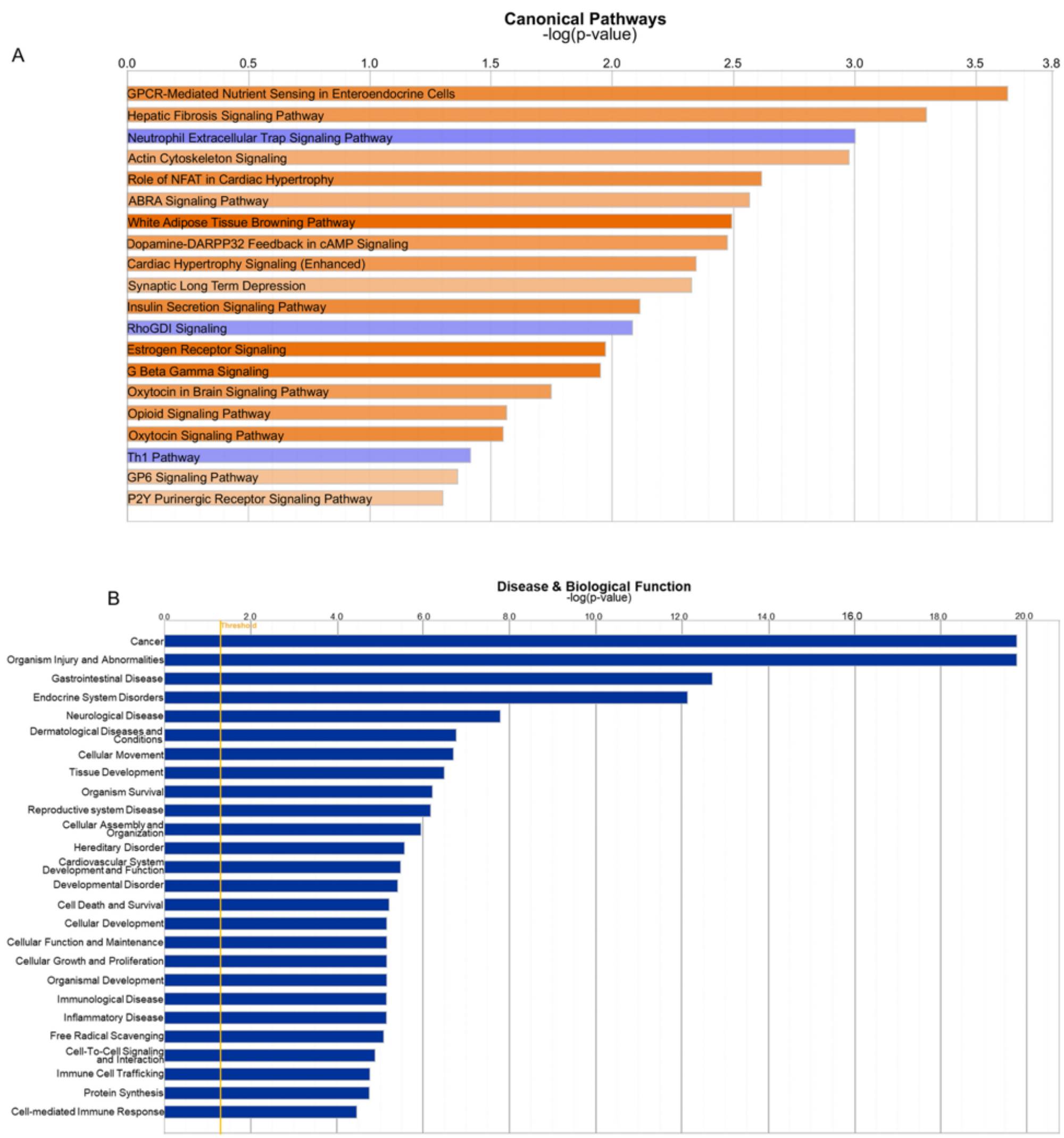

Supplementary Figure 4.

Effects of brief prior EV exposure on lung transcriptional responses to respiratory viruses in hamsters. A. Heatmap of canonical pathways significantly altered in lungs of hamsters by EV exposure prior to indicated respiratory viral infections compared to respective respiratory infections alone. Color denotations: blue=inhibition, orange=activation, white = no significant change. Arrows indicate: black: pathways similarly affected in the two groups; grey: pathways further explored in B-D. red: pathways uniquely activated by EV when exposure was followed by two viral infections. **B**. IFN signaling genes were significantly impacted in the lungs of hamsters by EV exposure prior to COV infection, compared to COV infection alone. Color denotations: blue=predicted inhibition, green = measured inhibition. **C-D**. Pathway of IL12 signaling affected by EV preexposure on COV- or on IAV and COV- induced lung effects.

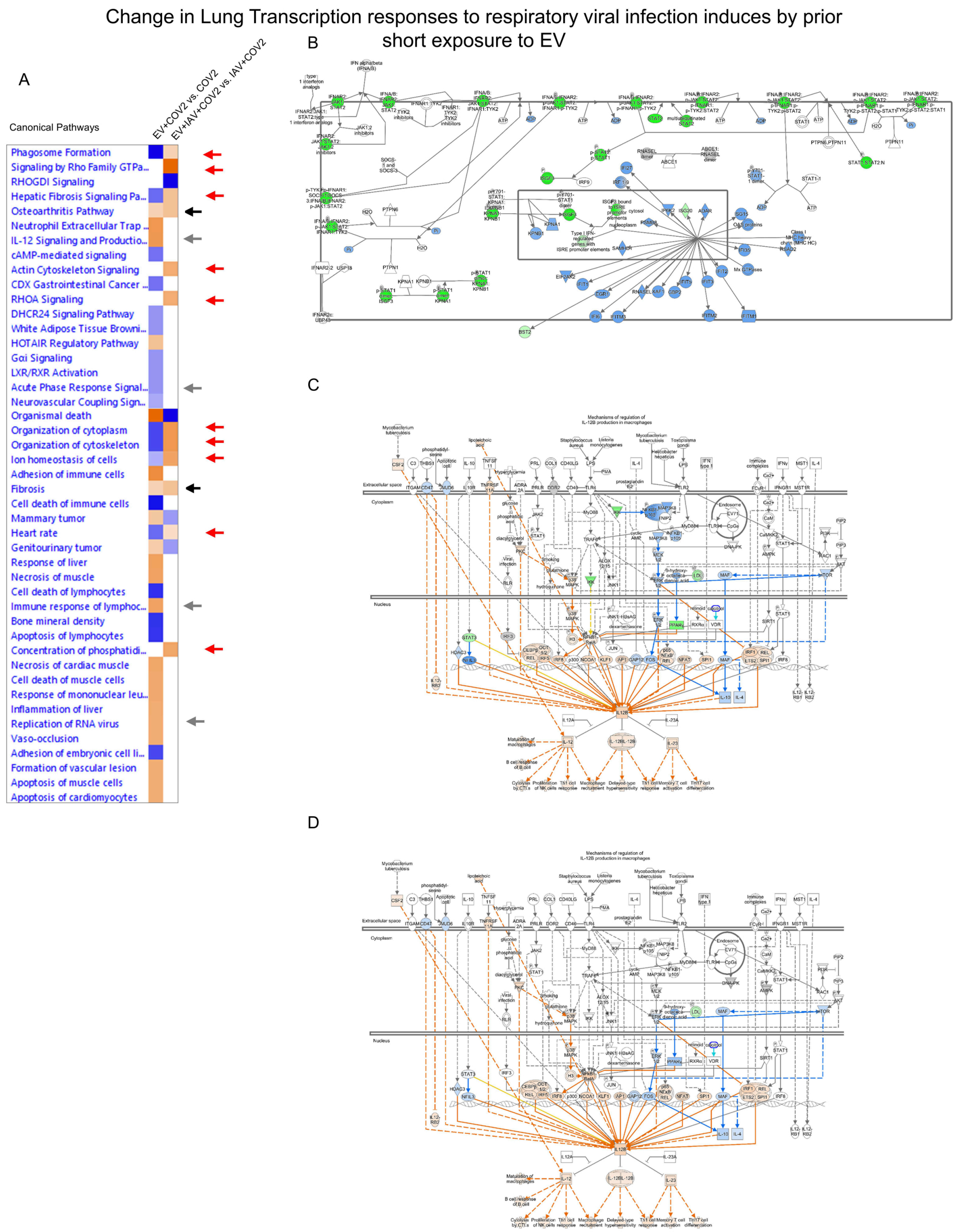

Supplementary Figure 5.

A. Heatmap of canonical pathways significantly altered in hamster lungs by IAV and COV-2 infection, IAV infection alone, and COV-2 infection alone, compared with uninfected (UT) animals. Color denotations: blue = inhibition, orange = activation, white = no significant change. Arrows indicate black: pathways specifically activated by IAV+COV-2. **B**. Bar graph with top-ranked pathways differentially altered in hamster lungs by COV-2 infection compared to lungs infected with IAV and then with COV-2. Bars are listed by -log(p-value), categorized into disease-related and cellular functional processes, with higher -log(p-value) values indicating stronger enrichment. The yellow threshold line indicates the cutoff for statistical significance. Blue indicates inhibition and orange indicates activation. **C-D.** Pathways of (C) IFN signaling and (D) estrogen signaling were significantly impacted in the lungs of hamsters by COV-2 infection compared to lungs previously infected with IAV and then with COV-2. Color denotations: orange = predicted increase; red = measured increase; blue = predicted decrease; green = measured decrease.

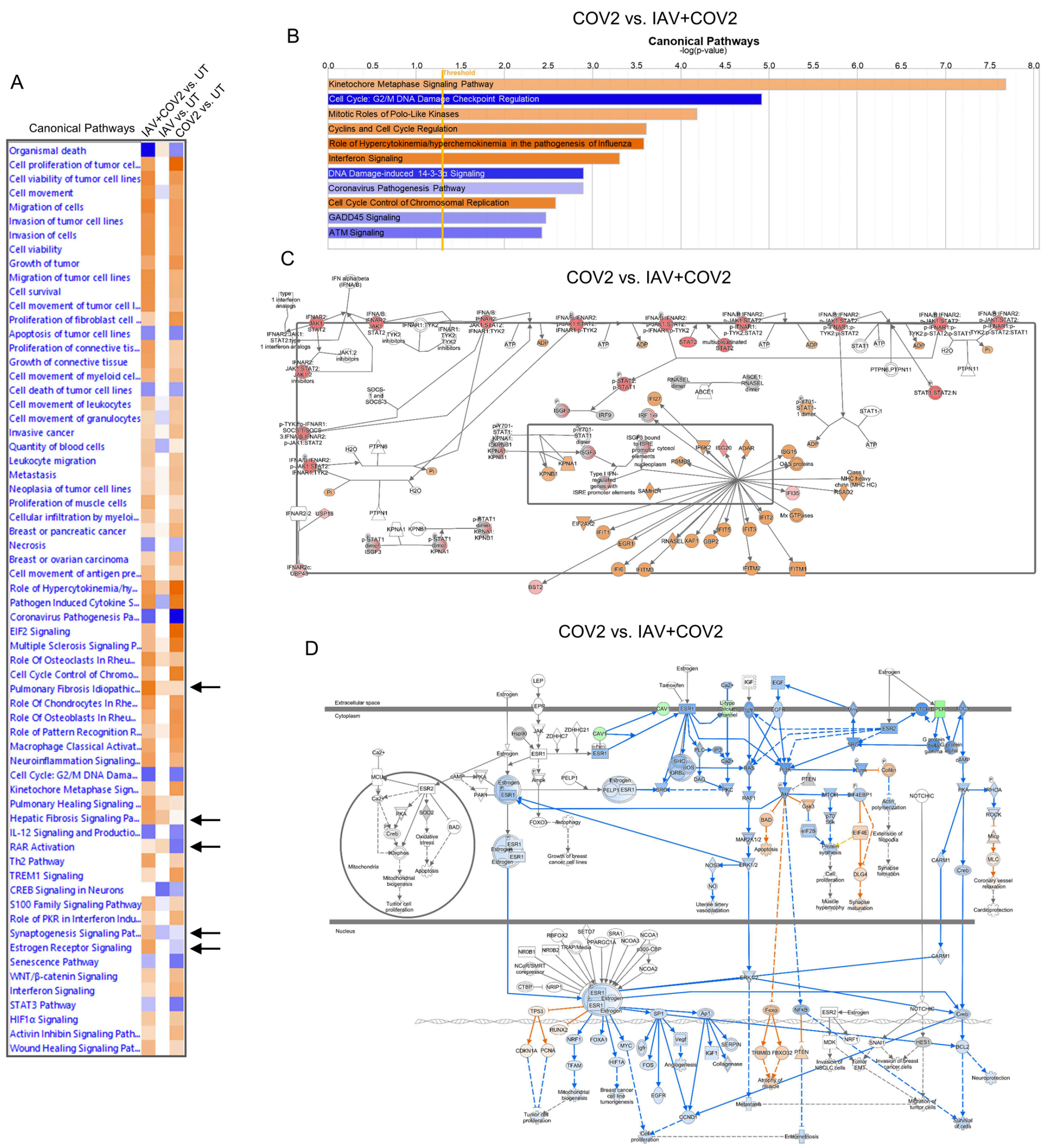

Supplementary Figure 6.

Characterization of small airway epithelial cell line. Representative immunofluorescence images of SAEC-KT grown at the air-liquid interface for 21 days and stained with differentiation markers: **A.** acetylated alpha-tubulin (green), CC10 (red), and nuclear staining with DAPI (blue). Yellow circles indicate ciliated cells. **B.** the differentiation marker KRT5 (green) and DAPI (blue). Yellow circles indicate basal cells.

A

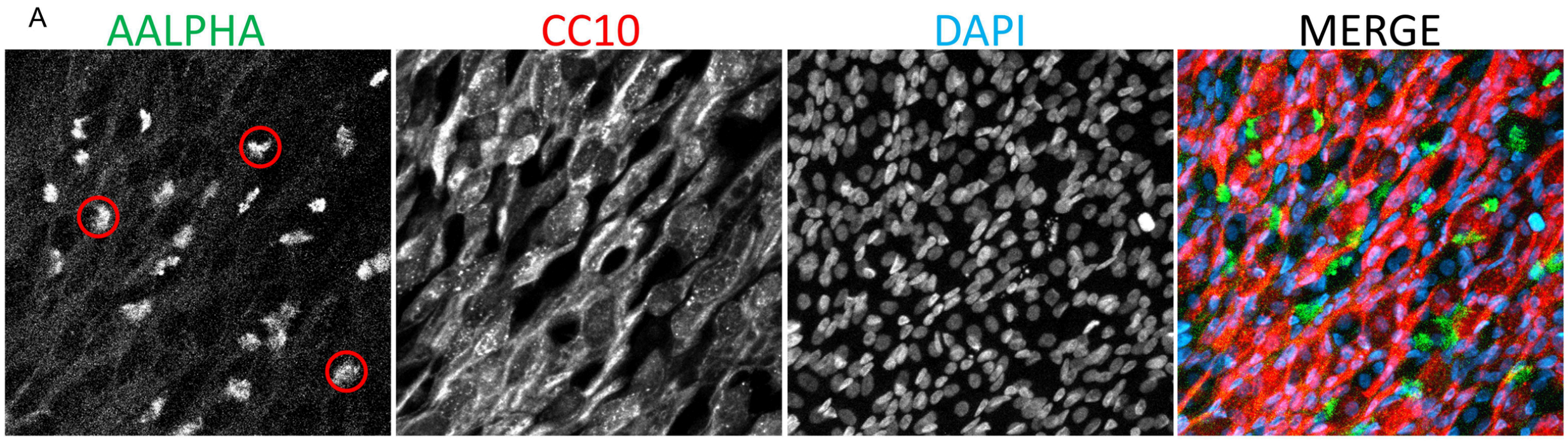

B

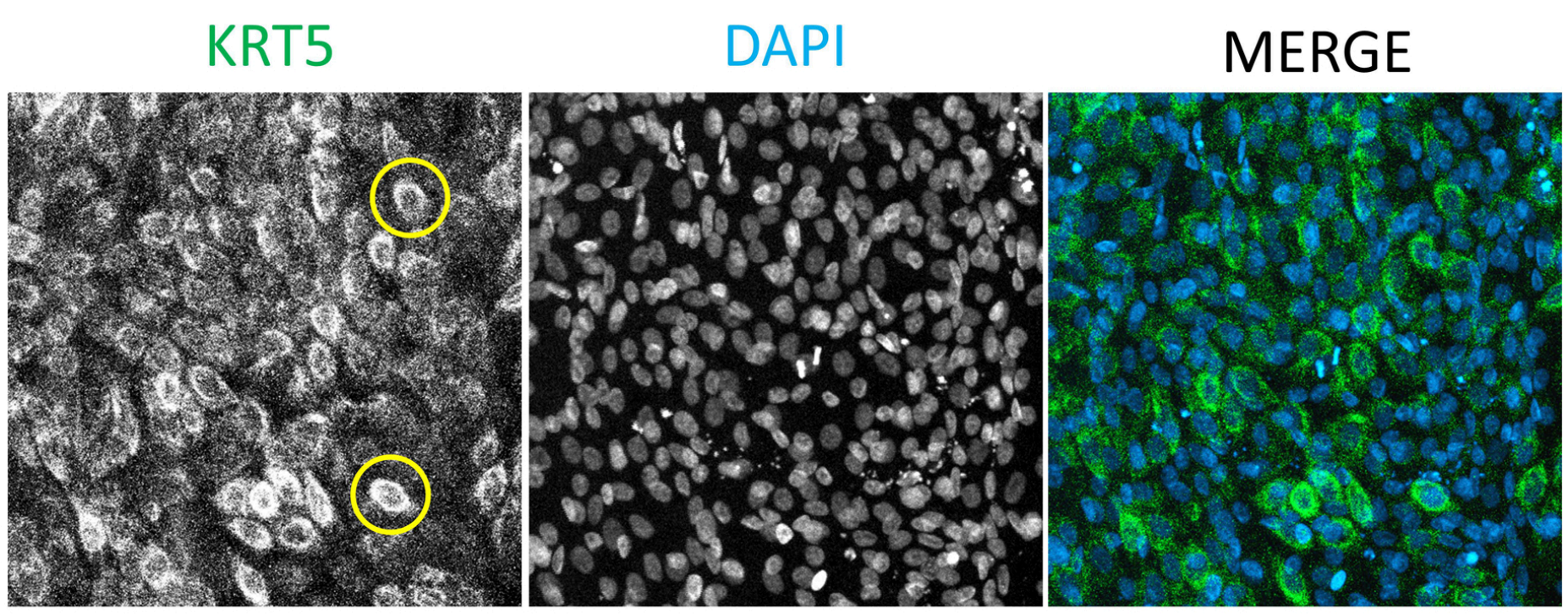

Supplement: Supplementary file 1 [file jciinsight-11-198757-s122.pdf]
