# Supplementary material for: E-cigarette exposure triggers distal lung cell injury, persistent lung stress response, and antiviral immune suppression
Source: JCI Insight. 2026 Aug 11;11(18):e198757. doi: 10.1172/jci.insight.198757 (PMC13596730; doi:10.1172/jci.insight.198757)
Supplement: Supplementary file 2 [file jciinsight-11-198757-s123.pdf]

Figure 2A Full Blot

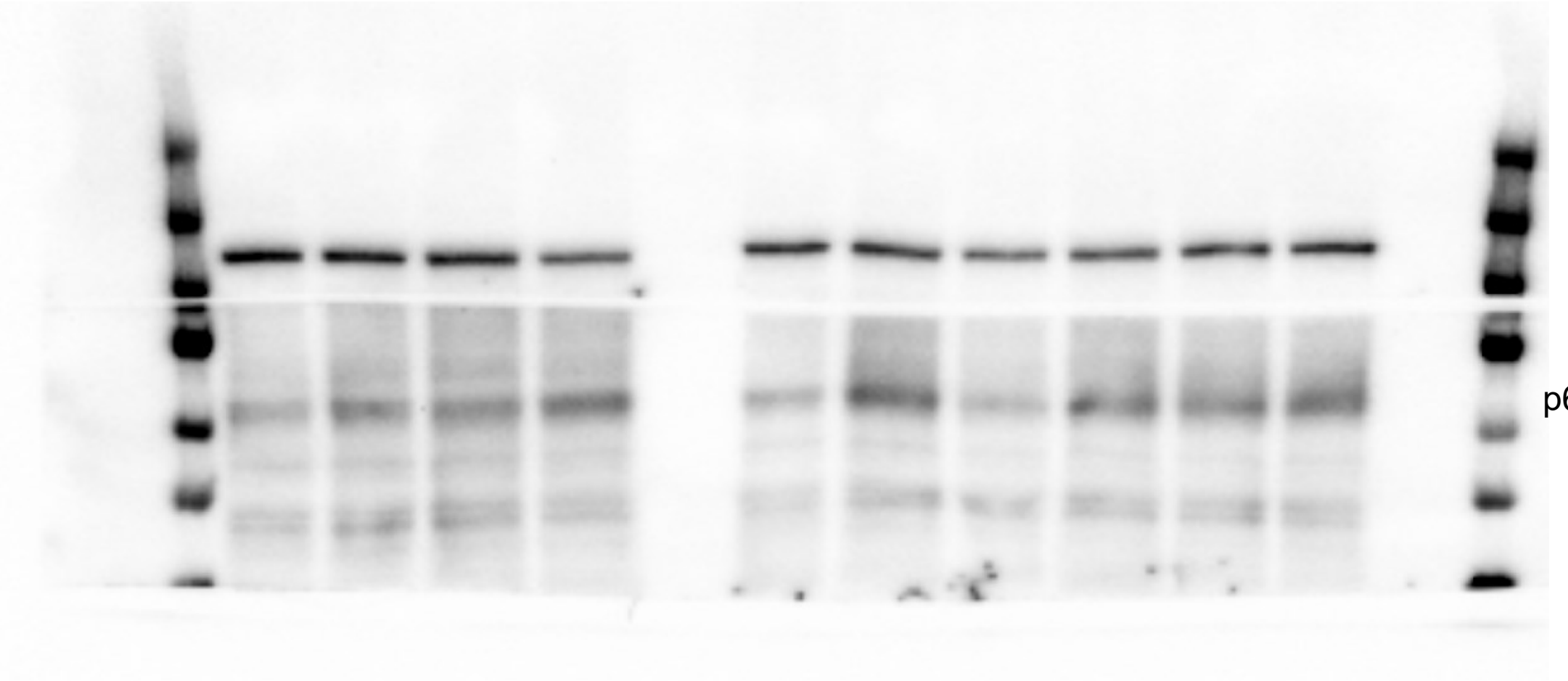

Vinculin (120 kDa)

p62 (62 kDa)

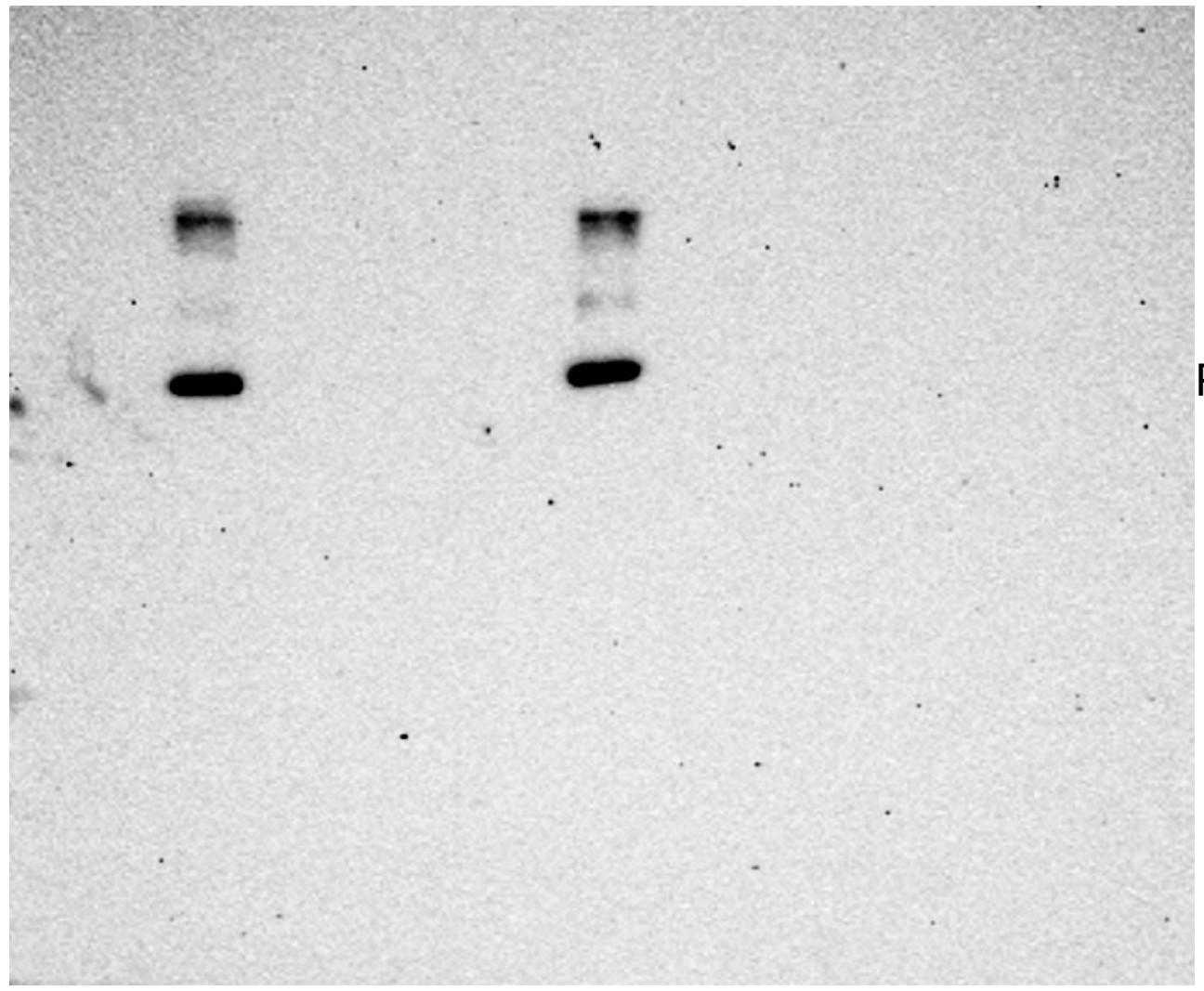

Phospho-S6 (30 kDa)

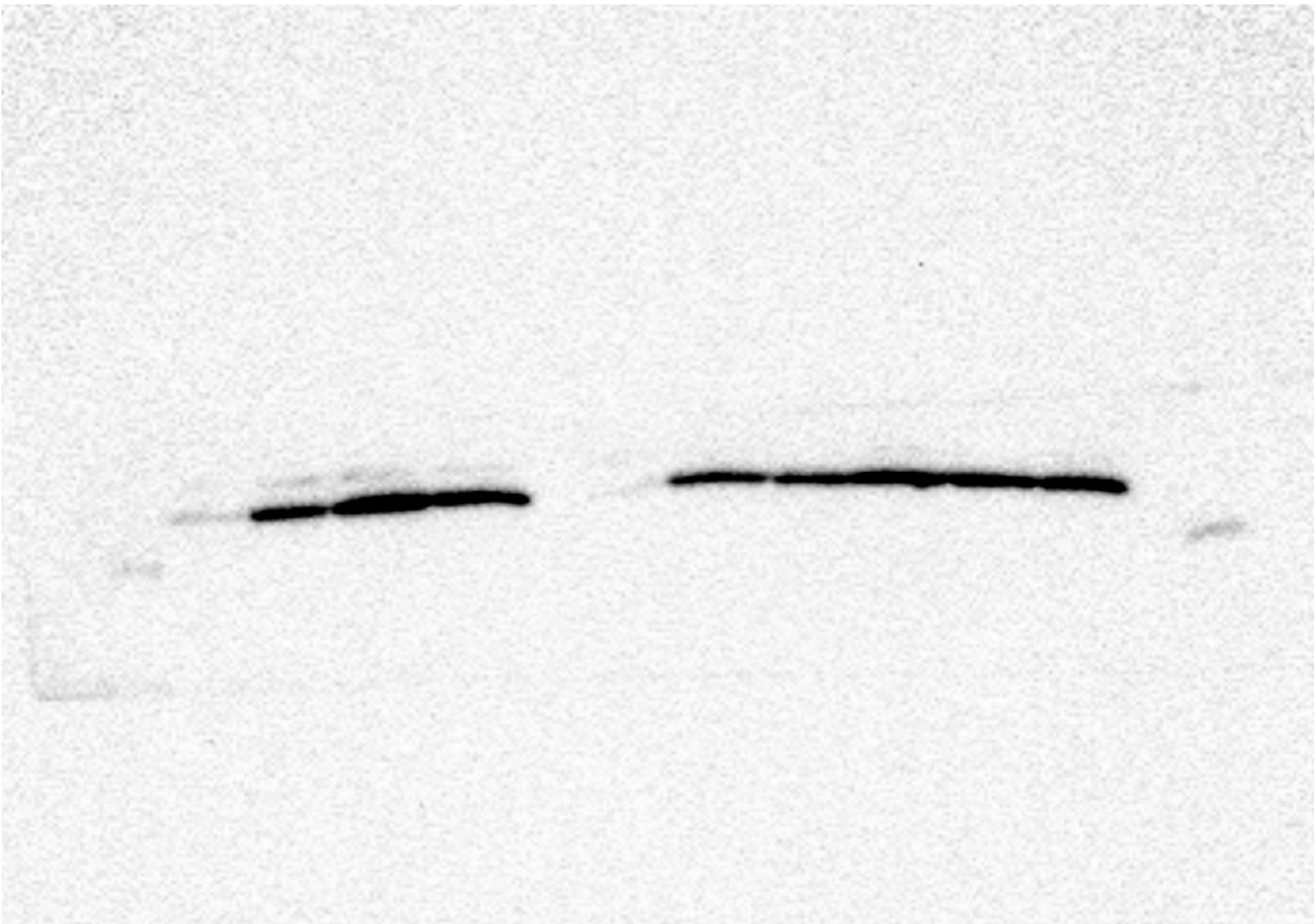

LC3B I & II (16-18kDa)

Figure 3A Full Blot

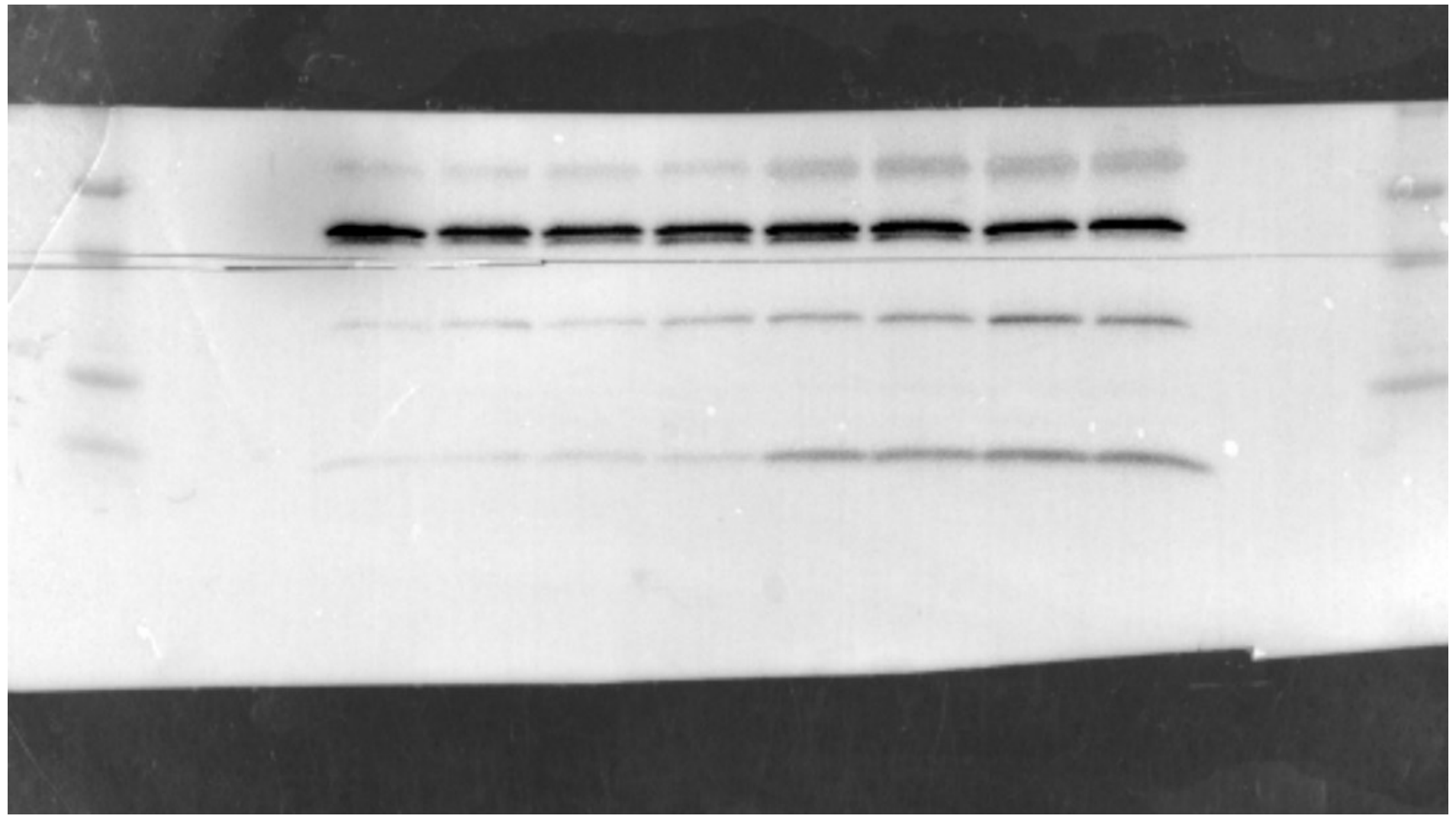

Beta Actin (42 kDa)

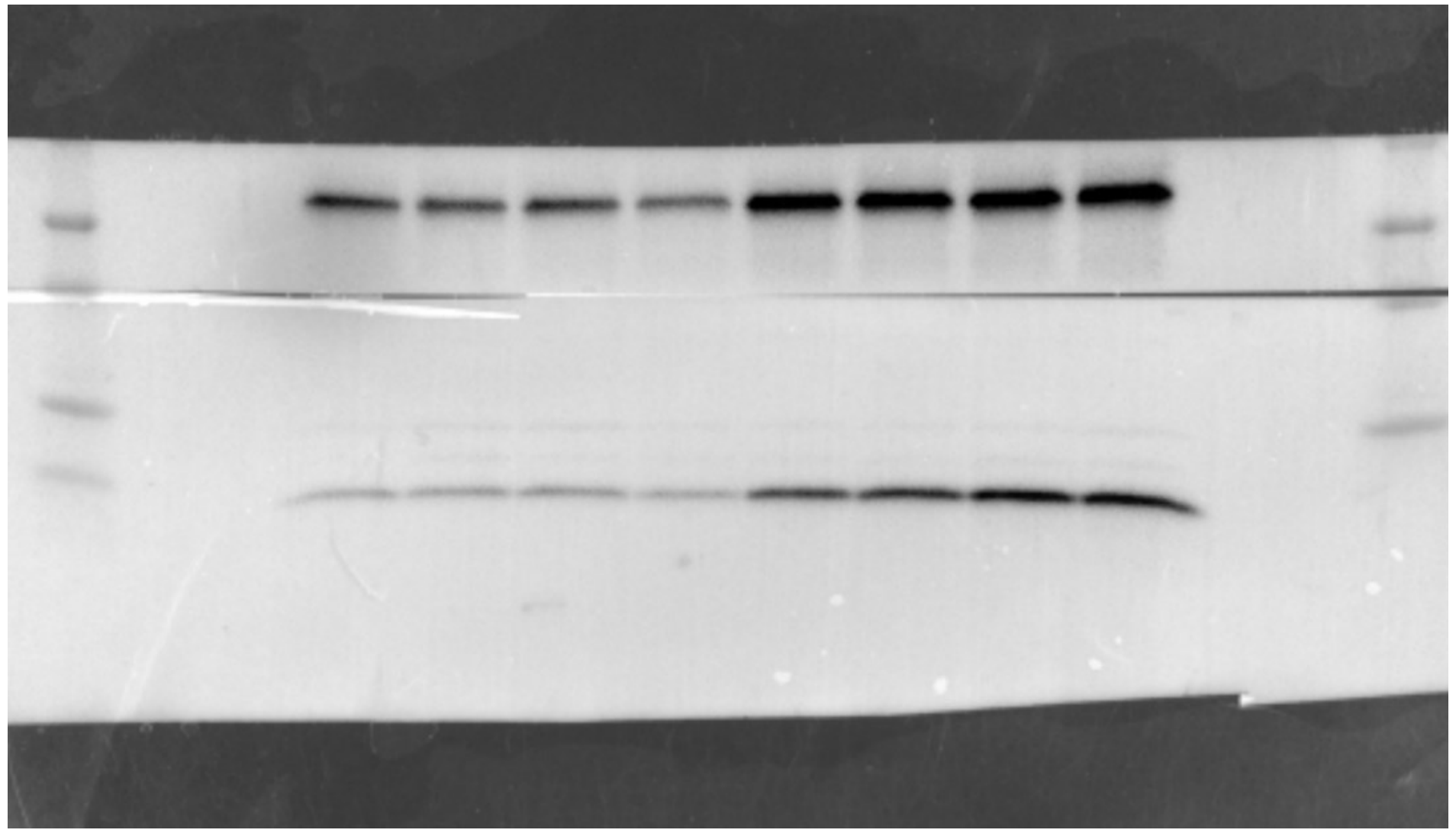

p62 (62 kDa)

LC3B II (16 kDa)

Figure 3I Full Blot

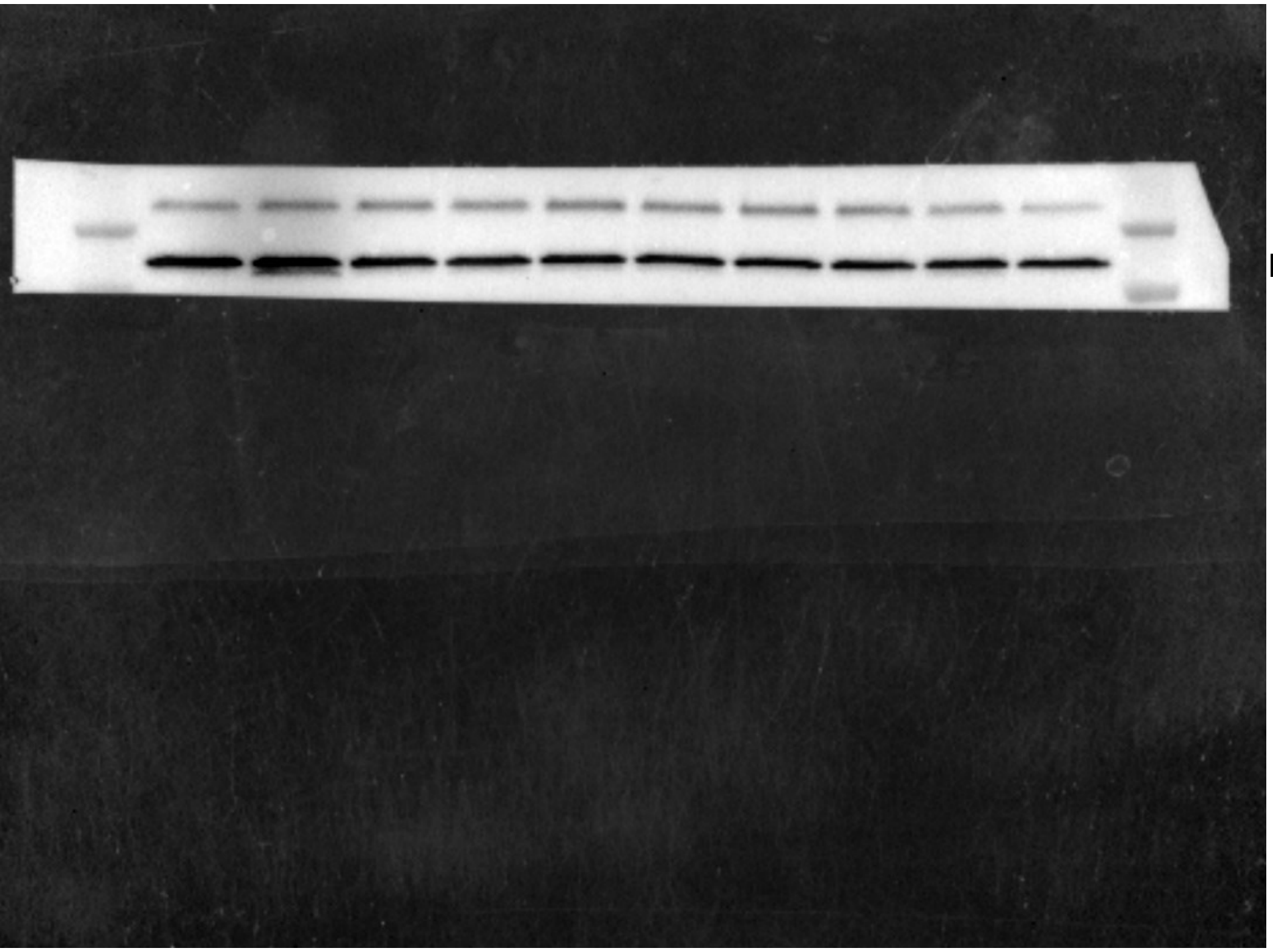

p62 (62 kDa)

Beta Actin (42 kDa)

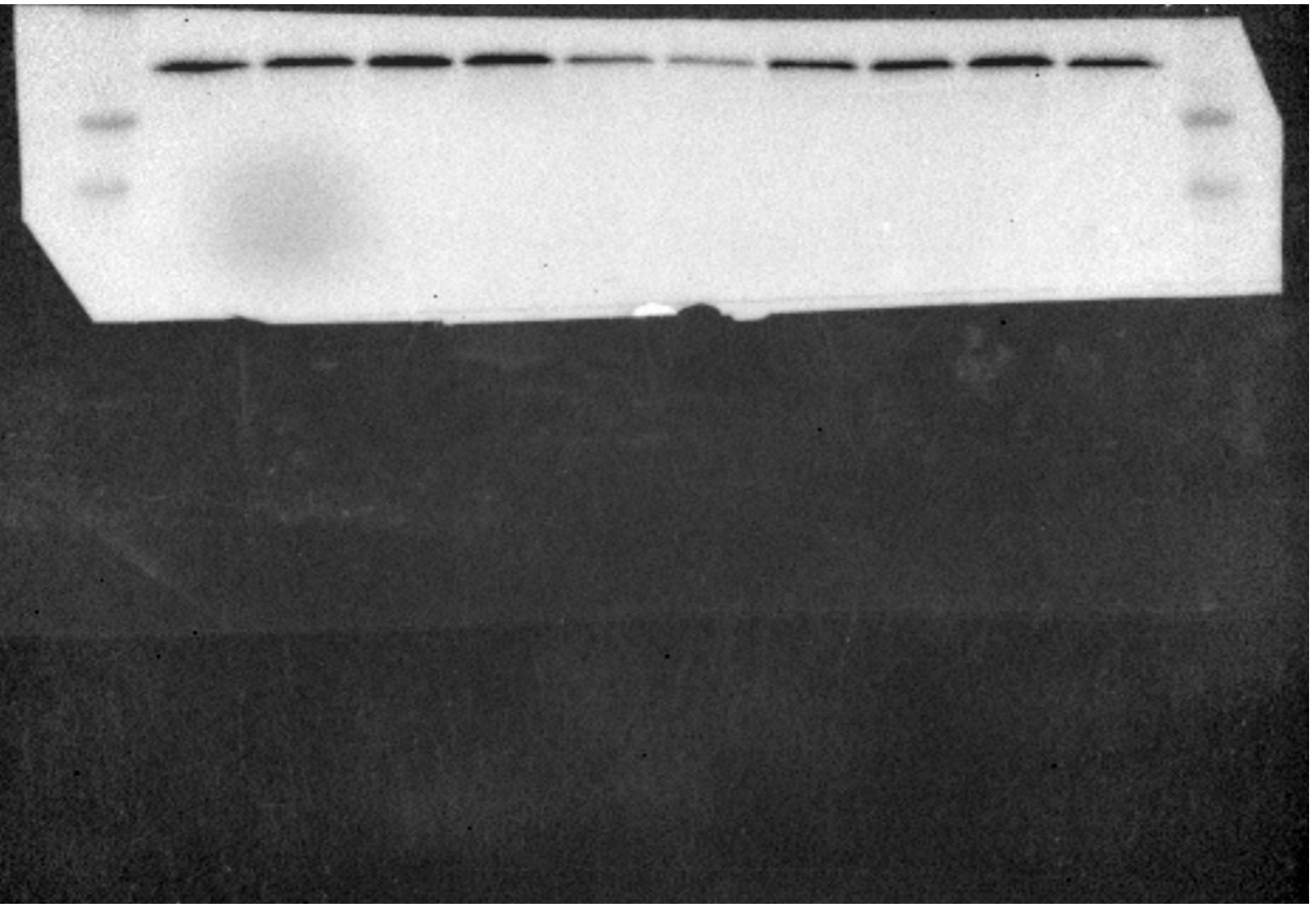

cParp (25 kDa)

Figure 3J Full Blot

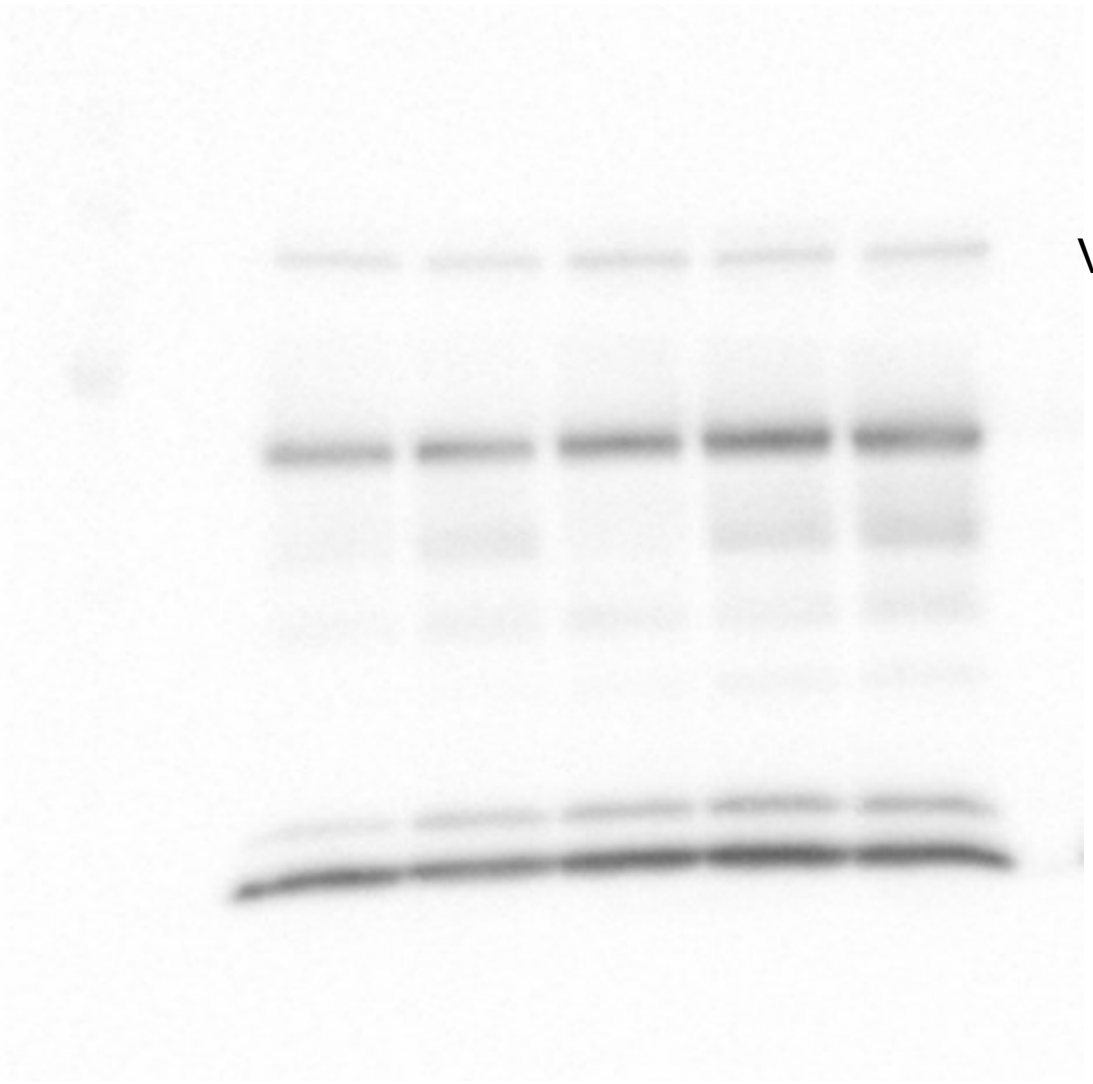

Figure 4D Full Blot

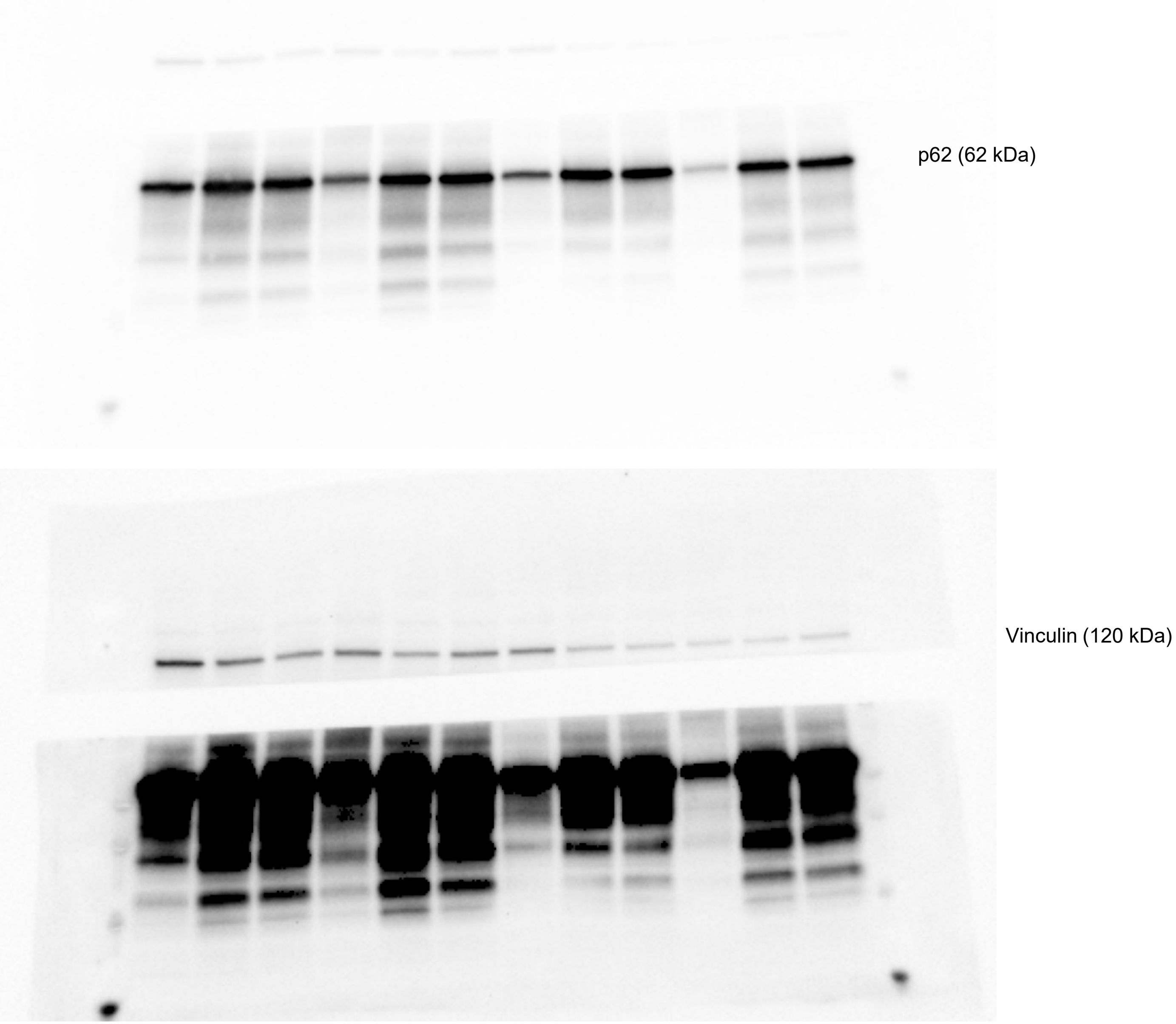

Figure 5B Full Blot

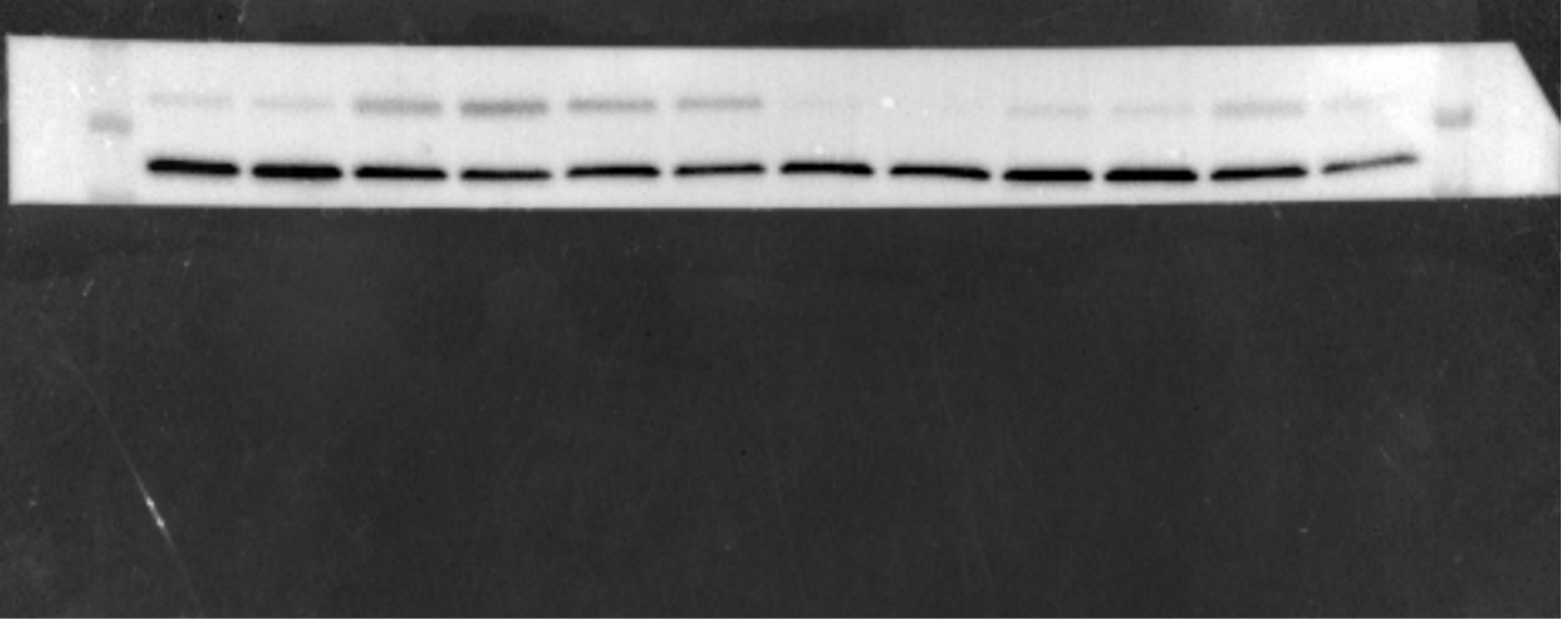

Beta Actin (42 kDa)

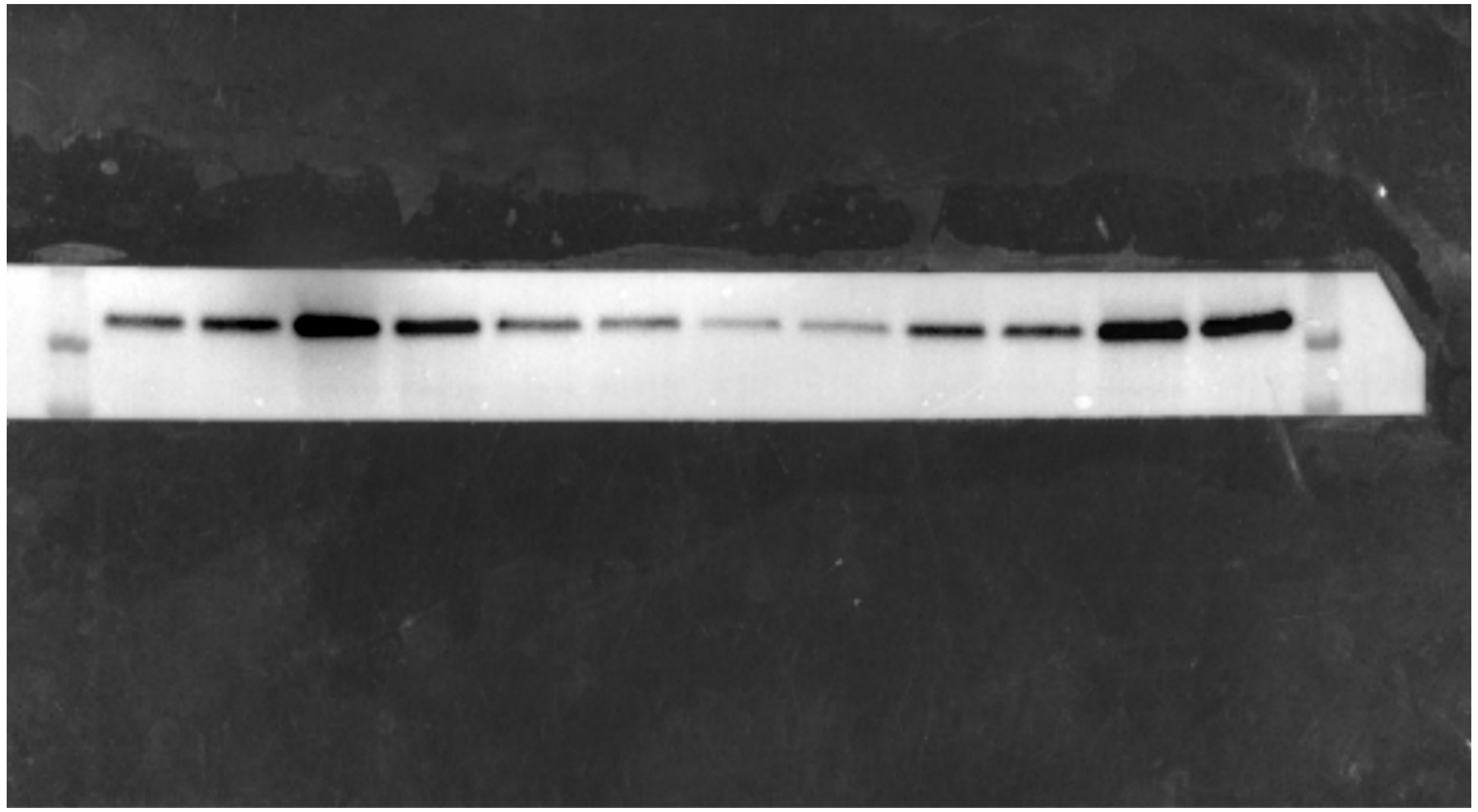

p62 (62 kDa)

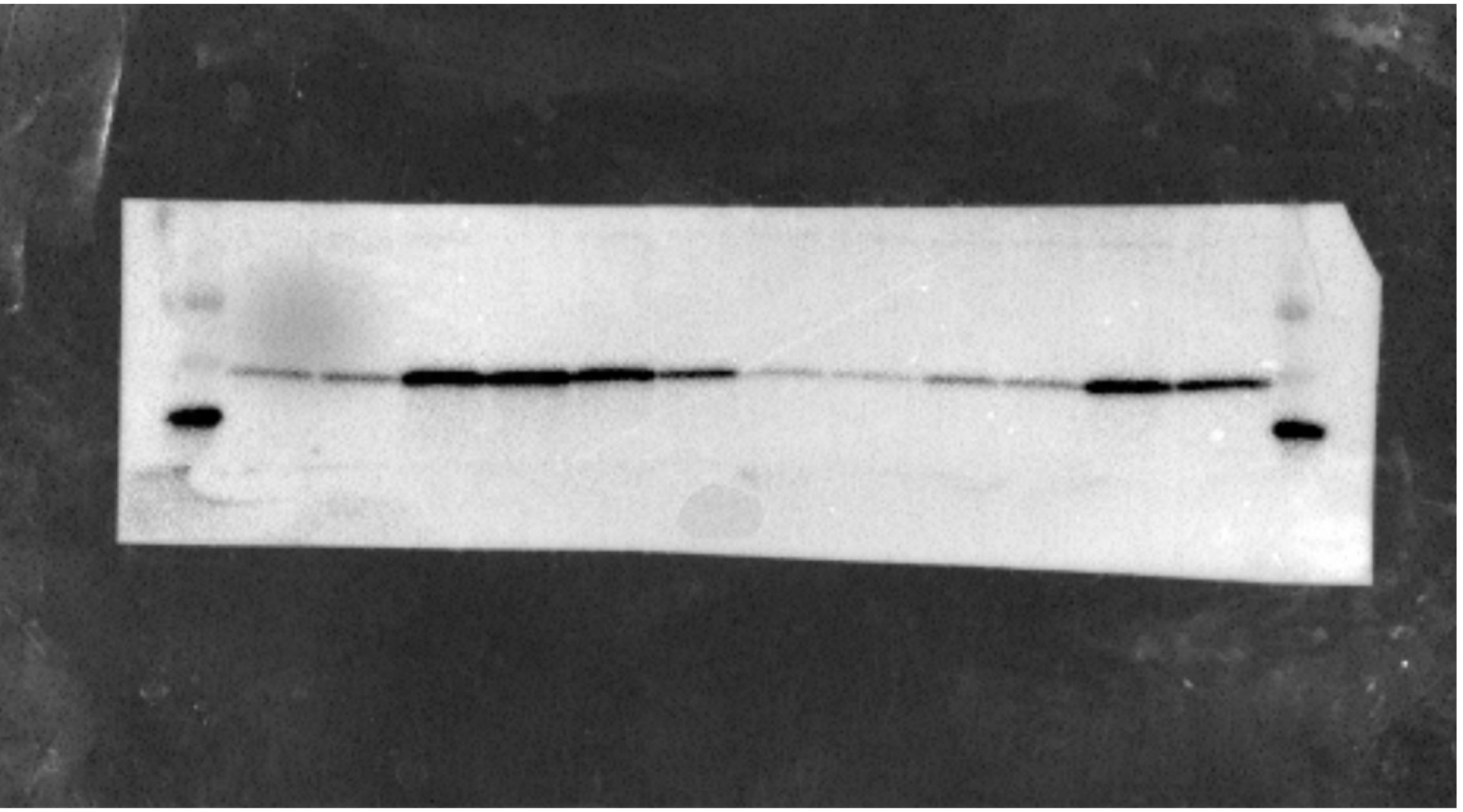

LC3B II (16 kDa)

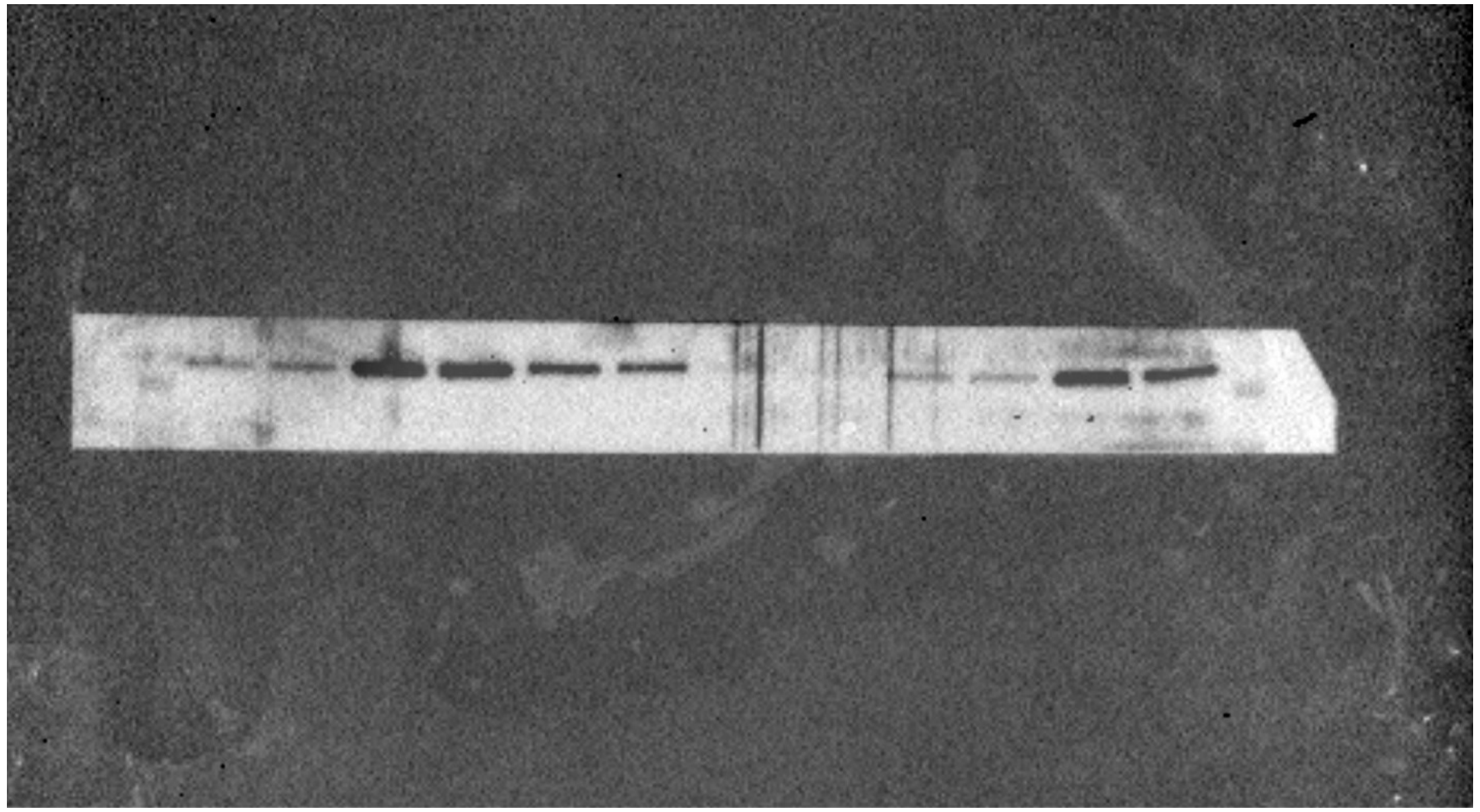

Phospho-JNK (46/54 kDa)

Figure 5F Full Blot

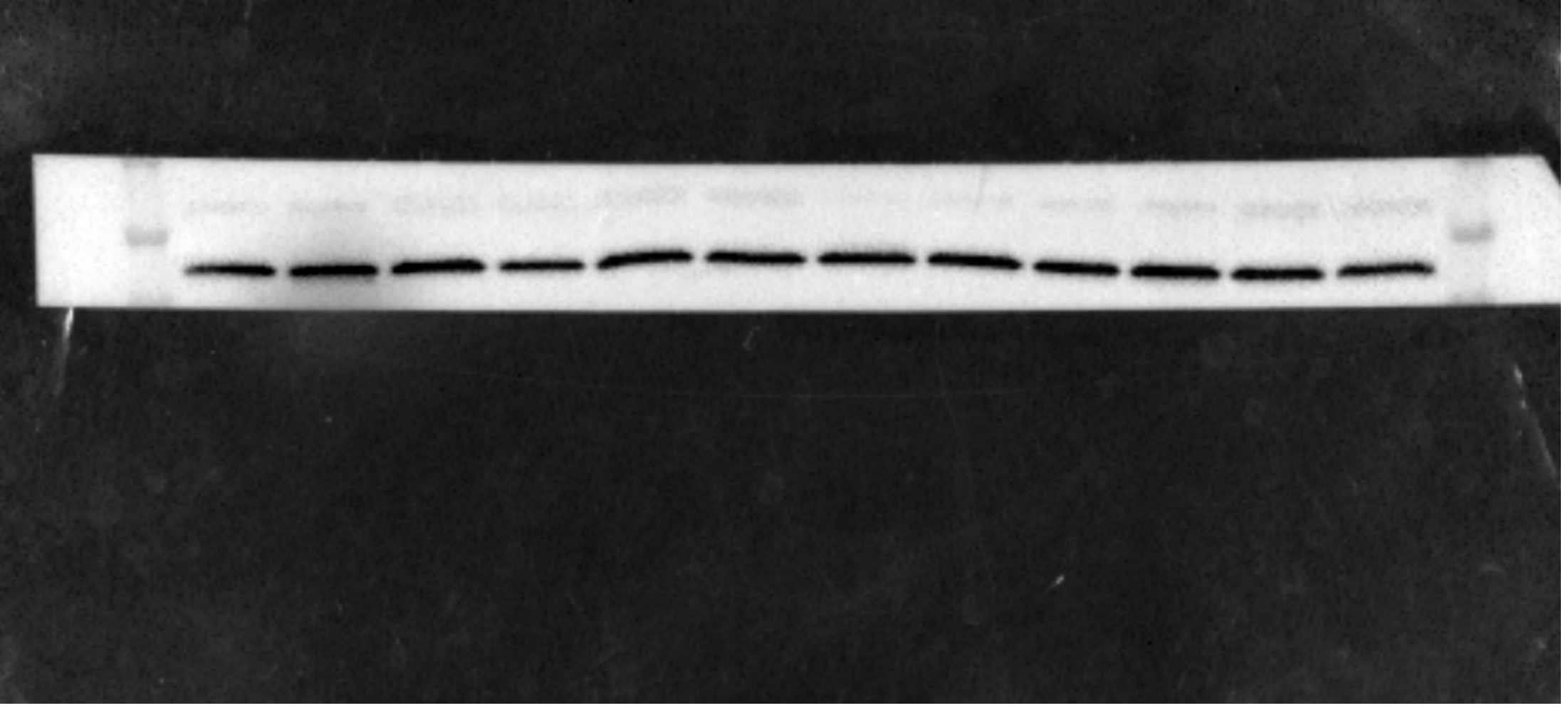

Beta Actin (42 kDa)

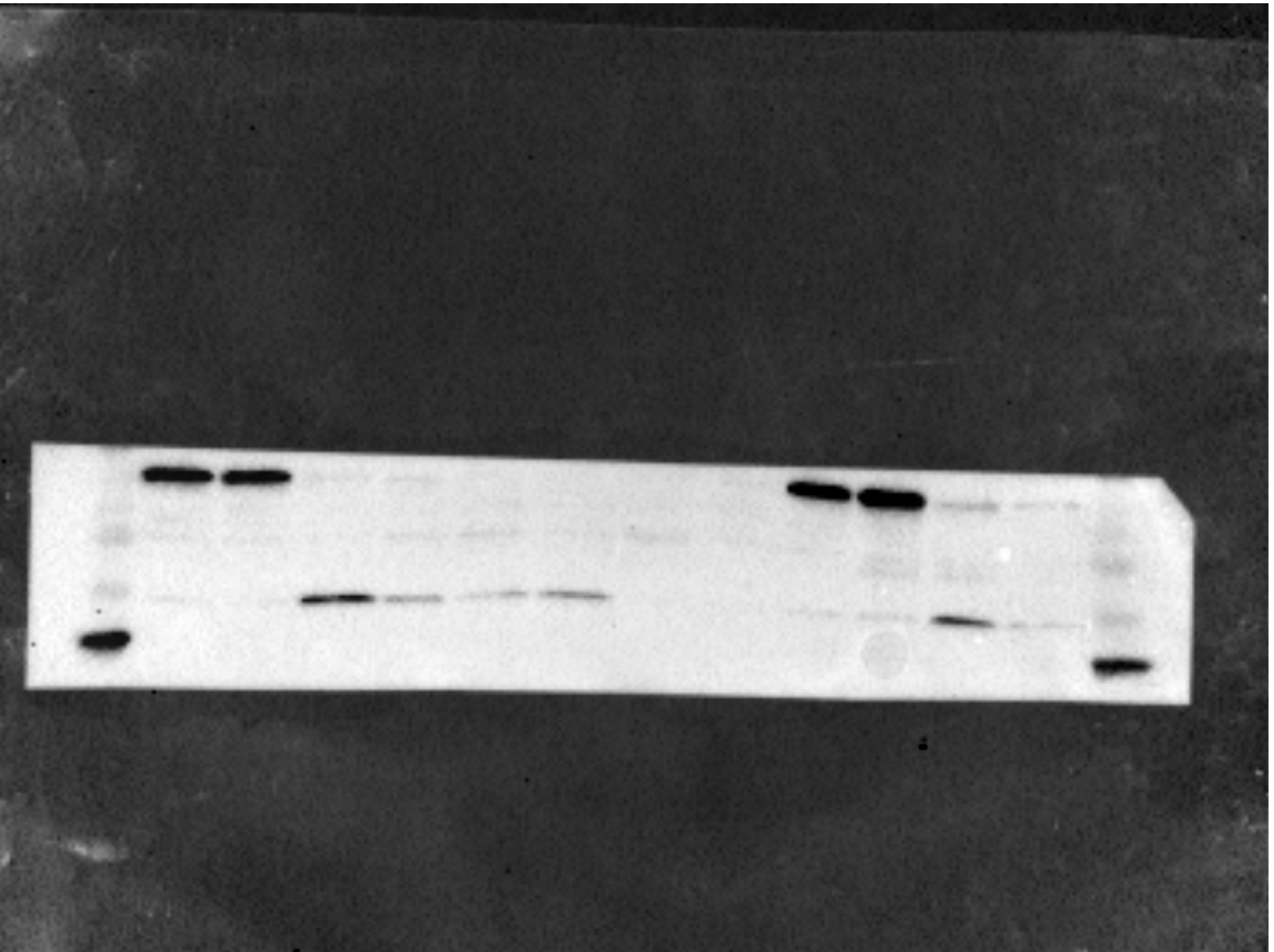

Phospho-S6 (30 kDa)
